# Targeting Thioredoxin-1 by dimethyl fumarate induces ripoptosome-mediated cell death

**DOI:** 10.1038/srep43168

**Published:** 2017-02-24

**Authors:** Anne Schroeder, Uwe Warnken, Daniel Röth, Karel D. Klika, Diana Vobis, Andrea Barnert, Fatmire Bujupi, Tina Oberacker, Martina Schnölzer, Jan P. Nicolay, Peter H. Krammer, Karsten Gülow

**Affiliations:** 1 Division of Immunogenetics, Tumor Immunology Program, German Cancer Research Center (DKFZ), Heidelberg, Germany; 2 Functional Proteome Analysis, German Cancer Research Center (DKFZ), Heidelberg, Germany; 3 Molecular Structure Analysis, German Cancer Research Center (DKFZ), Heidelberg, Germany; 4 Department of Dermatology, Venereology and Allergy, University Medical Center Mannheim, Mannheim, Germany

## Abstract

Constitutively active NFκB promotes survival of many cancers, especially T-cell lymphomas and leukemias by upregulating antiapoptotic proteins such as inhibitors of apoptosis (IAPs) and FLICE-like inhibitory proteins (cFLIPs). IAPs and cFLIPs negatively regulate the ripoptosome, which mediates cell death in an apoptotic or necroptotic manner. Here, we demonstrate for the first time, that DMF antagonizes NFκB by suppressing Thioredoxin-1 (Trx1), a major regulator of NFκB transcriptional activity. DMF-mediated inhibition of NFκB causes ripoptosome formation *via* downregulation of IAPs and cFLIPs. In addition, DMF promotes mitochondrial Smac release and subsequent degradation of IAPs, further enhancing cell death in tumor cells displaying constitutive NFκB activity. Significantly, CTCL patients treated with DMF display substantial ripoptosome formation and caspase-3 cleavage in T-cells. DMF induces cell death predominantly in malignant or activated T-cells. Further, we show that malignant T-cells can die by both apoptosis and necroptosis, in contrast to resting T-cells, which are restricted to apoptosis upon DMF administration. In summary, our data provide new mechanistic insight in the regulation of cell death by targeting NFκB *via* Trx1 in cancer. Thus, interference with Trx1 activity is a novel approach for treatment of NFκB-dependent tumors.

Nuclear factor-κB (NFκB) is a central transcription factor orchestrating innate and adaptive immune responses. In acute inflammation, NFκB activity is tightly regulated. However, aberrantly activated NFκB is associated with chronic inflammatory diseases and a variety of human cancers including both solid and hematopoietic malignancies. Cancers such as T-cell acute lymphoblastic leukemia (T-ALL), cutaneous T-cell lymphoma (CTCL), and its leukemic variant, Sézary Syndrome, revealed constitutive NFκB activity^1^,^2^,^3^,^4^.

The NFκB family consists of five Rel related proteins: RelA (p65), RelB, cRel, p50 and p52, which can form both homo- and heterodimers. The typical NFκB complex is a p65/p50 heterodimer critical for NFκB mediated anti-apoptotic effects^5^. In its inactive form, NFκB is sequestered in the cytoplasm by IκBα. Phosphorylation and proteasomal degradation of IκBα releases NFκB. Subsequent nuclear translocation and full activation of NFκB is redox-dependent and mediated by phosphorylation^6^. The redox regulator Thioredoxin-1 (Trx1) promotes DNA binding activity of NFκB by reduction of a cysteine residue within its DNA binding domain^7^,^8^. During oncogenesis, NFκB promotes cell survival and proliferation by inducing expression of molecules associated with suppression of programmed cell death (PCD), such as cFLIPs^9^, IAP proteins^6^,^10^, and members of the Bcl-2 family^11^. PCD is a mechanism of tumor suppression and manifests itself in, *e.g.* apoptosis and necroptosis. Necroptosis is a form of regulated necrosis, which has been implicated to trigger strong immune responses by release of damage-associated molecular patterns (DAMPs)^12^. Moreover, necroptosis is critical for T-cell homeostasis as backup to eliminate an excess of activated T-cells after clonal expansion preventing autoimmunity^13^.

The ripoptosome is a signaling platform triggering cell death in an apoptotic or necroptotic manner^14^,^15^,^16^. The core components of the ripoptosome include caspase-8, FADD (Fas-associated death domain) and RIPK1 (Receptor-interacting kinase 1). Formation and activation of the ripoptosome are negatively regulated by IAPs (cIAP1, cIAP2 and XIAP) and cFLIPs (cFLIP_L_ and cFLIP_S_), respectively. IAPs are regulated by Smac (Second mitochondria-derived activator of caspases) released by mitochondria in response to pro-apoptotic stimuli. In the cytosol, Smac interacts and antagonizes IAPs. MOMP (mitochondrial outer membrane permeabilization)-associated Smac release is regulated by Bcl-2 family members^17^. The caspase-8 regulators cFLIPs modulate the ripoptosome response. While cFLIP_L_ seems to suppress ripoptosome activity, overexpression of cFLIP_S_ diminishes caspase-8 activity, thus, promoting necroptosis^15^. Notably, ripoptosome formation predominantly occurs in malignant cells^16^.

Evasion from PCD is a hallmark of cancer and facilitates immune escape, chemoresistance and poor prognosis. Regulators of PCD, such as IAPs, are frequently overexpressed in many cancer cells. Therefore, it is of great interest to design novel therapeutics targeting cell death resistant cancer cells. So far, several small molecule inhibitors have been developed to facilitate depletion of IAPs. Smac mimetics bind to IAPs leading to rapid auto-ubiquitylation and degradation^18^. Depletion of IAPs may also occur by chemotherapeutic drugs, which induce genotoxic stress such as etoposide^19^. Since IAPs, cFLIPs and Bcl-2 family members are target genes of NFκB, NFκB is an attractive target for cancer therapy. Clinically DMF is a promising therapeutic agent for CTCL since DMF has limited side effects compared to other NFκB inhibitors, which display relatively high toxicity^2^,^20^. However, the exact molecular mechanism of DMF-induced NFκB inhibition and subsequent cell death remains to be elucidated.

Here, we show that DMF (Tecfidera^®^), a FDA-approved drug for treatment of multiple sclerosis, blocks Trx1 activity by modification of a specific thiol group. Reduced Trx1 activity leads to inhibition of NFκB. Remarkably, DMF-mediated inhibition of the Trx1/NFκB axis results in ripoptosome formation and subsequent PCD by downregulation of cIAP2 and cFLIPs *in vitro* and *in vivo*. Full ripoptosome activation requires mitochondria and release of Smac. Cytosolic Smac promotes degradation of cIAP1 and XIAP, further enhancing ripoptosome formation and activation in an amplification loop. Moreover, DMF treated Sézary patients reveal ripoptosome assembly which coincides with caspase-3 cleavage. DMF administration induced cell death more efficiently in malignant CD4^+^ T-cells isolated from Sézary patients compared to resting T-cells. Other than apoptosis which is induced in T-cells from Sézary patients and healthy donors, necroptosis predominantly occurs in malignant T-cells.

Hence, targeting the Trx1/NFκB signal transduction by DMF may represent a promising treatment for lymphoproliferative diseases. Our data further provide a rationale for the development of novel compounds treating NFκB dependent tumors.

## Results

### DMF suppresses activity of Trx1 by monomethyl succinylation

DMF is a potent inhibitor of NFκB^21^, though the molecular mechanism by which DMF suppresses transcriptional activity of NFκB is unclear. DMF has been shown to modify reactive thiol groups in *e.g.* glutathione (Supplementary Data and Supplementary Fig. S1A)^22^. Together with glutathione, thioredoxin proteins (Trx1 and Trx2) control cellular reactive oxygen species (ROS). In addition, Trx1 controls the redox state of cysteine residues in proteins such as NFκB^23^. Therefore, we set out to determine whether inhibition of NFκB is mediated by DMF-dependent suppression of Trx1.

**Figure 1:**
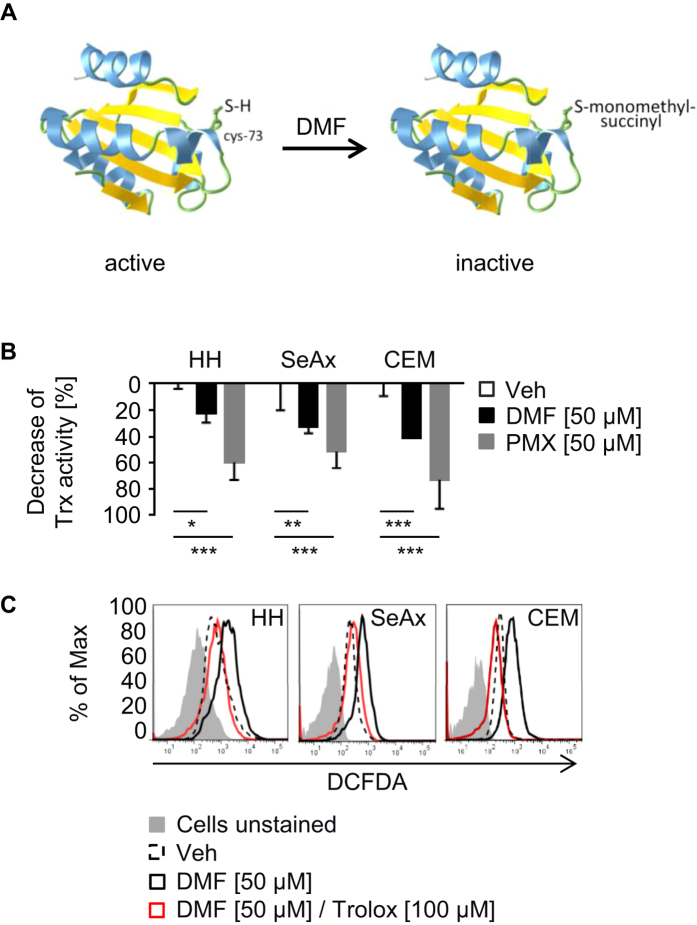
DMF inhibits Trx1 activity. (**A**) Trx1 is monomethyl succinylated at cysteine 73 (C73). To generate the 3D structure of Trx1 Aquaria database software (http://aquaria.ws/) was used^49^,^50^. (**B**) Trx activity of cells was measured 2 h after treatment with DMSO (Veh), DMF, or PMX464 (PMX) (n = 3). (**C**) HH, SeAx and CEM cells were left unstimulated or stimulated with DMSO (Veh), DMF or DMF and Trolox for 2 h. Untreated cells were left unstained. Treated cells were stained with H_2_DCFDA for 30 min and analyzed by flow cytometry. (**B**) Error bars represent standard deviation. Statistics were calculated using Student’s t-test (*p < 0.05; **p < 0.005; ***p < 0.001).

Mass spectrometry (MS) was used to identify chemical modifications of Trx1 in response to DMF. Interestingly, the redox sensitive cysteines (C32 and C35) have not been targeted. However, C73, which possesses a regulatory function controlling general activity of Trx1^24^,^25^,^26^,^27^, was identified as monomethyl succinylated (Fig. 1A, Supplementary Fig. S1B–D). Notably, we detected no modification of Trx2. Equal concentrations of MMF (25 μM), a hydrolization product of DMF, did not result in detectable modifications of Trx1 (data not shown).

To investigate the impact of monomethyl succinylation activity of Trx1 was determined. Modification of Trx1 led to an inhibition of its activity. PMX464, a known Trx inhibitor^28^, was used as positive control (Fig. 1B). Furthermore, DMF-dependent suppression of Trx1 resulted in elevated levels of ROS. Treatment with trolox, a water-soluble vitamin E derivative and thiol-independent antioxidant, blocked DMF-dependent ROS accumulation (Fig. 1C). Thus, monomethyl succinylation of C73 by DMF inhibits Trx1 activity.

### NFκB inhibition by DMF induces loss of cIAP2 and cFLIP

**Figure 2:**
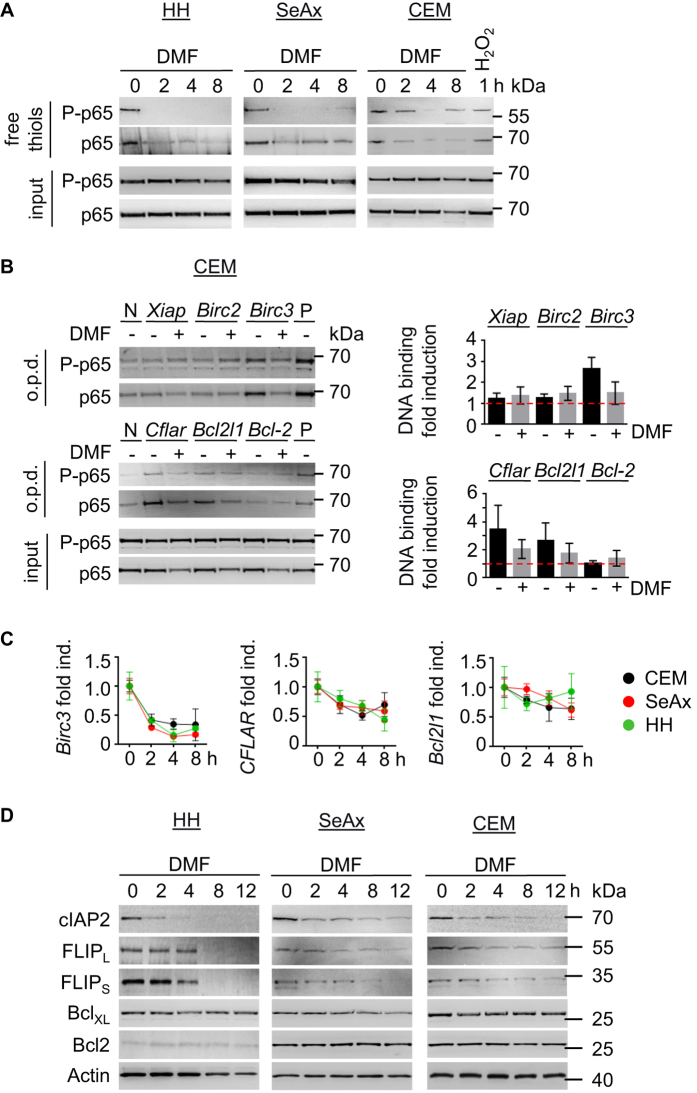
DMF-dependent modification of Trx leads to inhibition of NFκB activity. (**A**) Cells were treated with DMF (50 μM) in a time-dependent manner as indicated. CEM cells were incubated with H_2_O_2_ (100 μM) for 2 h as positive control. Reduced proteins were pulled-down using biotinylated iodoacetamide (BIAM) and probed for phospho- and total NFκB. (**B**) CEM cells were left untreated or treated with DMF (50 μM) for 4 h. NFκB DNA binding was assessed by oligo pull-down (o.p.d.) for indicated biotinylated oligonucleotides containing NFκB binding sites. Positive and negative controls are denoted by P or N respectively (left). Band intensities were quantified from 3 independent experiments and normalized to negative control (right). (**C**) Quantitative real-time PCR of indicated genes was performed in triplicates using RNA derived from cells treated with DMF (50 μM) for 2, 4, and 8 h. (**D**) HH, SeAx and CEM cells were incubated with DMF (50 μM) for the indicated time points. Cell lysastes were analyzed by Western blot. (**B,C**) Error bars represent standard deviation.

As a regulator of NFκB transcriptional activity Trx1 is an attractive target for cancer therapy. Trx1 promotes transcriptional activity of NFκB by reducing a cysteine in its DNA binding domain^23^. To evaluate the redox state of NFκB upon DMF treatment, proteins containing free thiols were precipitated. Inhibition of Trx1 in response to DMF led to a time-dependent accumulation of inactive/oxidized NFκB. Cysteines in NFκB were oxidized 2 h after DMF treatment (Fig. 2A). Similarly, PMX464 suppressed reduction of cysteines in NFκB (Supplementary Fig. S2A).

Oxidized cysteines in NFκB should result in diminished DNA binding. Therefore, we tested DNA binding of NFκB on target genes such as *Birc2, Birc3, Xiap, Cflar, Bcl-2* and *Bcl2l1*. DNA oligonucleotides with NFκB binding sites were pulled-down and probed for the presence of NFκB. DNA binding of NFκB to oligonucleotides of *Birc2, XIAP* and *Bcl-2* was not regulated in response to DMF. However, DMF treatment suppressed NFκB binding to *Birc3, Cflar* and *Bcl2l1* (Fig. 2B; Supplementary Fig. S2B). Consistently, DMF inhibited mRNA and protein expression of cIAP2 and cFLIPs (Fig. 2C,D). However, Bcl_XL_ expression was only moderately impaired on mRNA and protein level (Fig. 2C,D). Bcl-2 was not regulated in response to DMF (Fig. 2D; Supplementary Fig. S2C). Collectively, our data demonstrate that inhibitors of Trx1 such as DMF and PMX464 suppress reduction of cysteines in NFκB. DMF-dependent oxidation of cysteines in NFκB results in diminished expression of cIAP2 and cFLIPs (Supplementary Fig. S2D).

### Inhibition of NFκB activity results in ripoptosome formation

**Figure 3:**
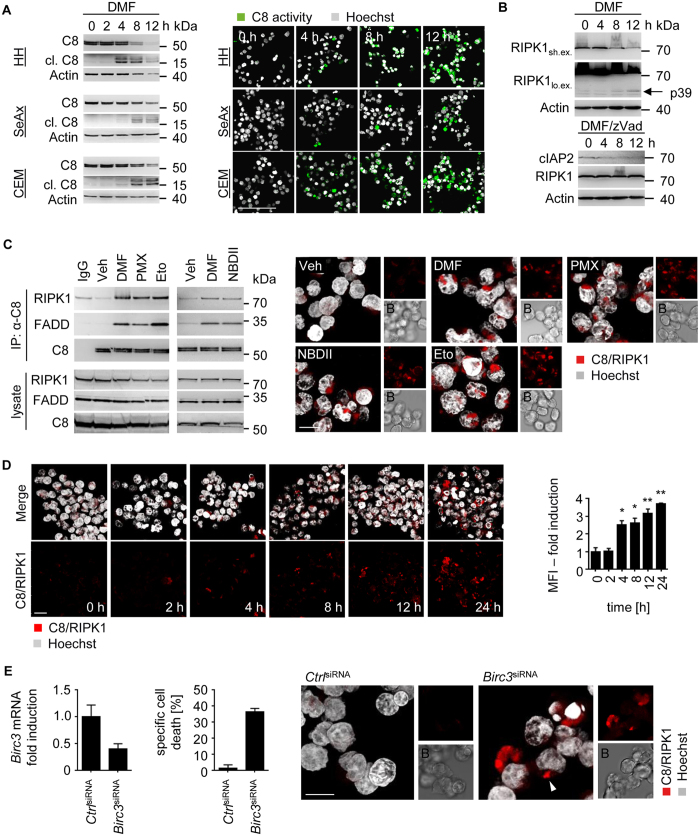
Ripoptosome formation induced by DMF results in caspase-8-dependent cell death. (**A**) CEM cells were treated with DMF (50 μM) for the indicated time points. Caspase-8 (C8) cleavage was determined by Western blot (left), immunofluorescence (IF) shows cleaved caspase-8; for IF, Hoechst (grey), caspase-8 cleavage (green); scale bar, 100 μm (right). (**B**) Western blot analysis of DMF (50 μM) or DMF/zVad treated CEM cells. Cells were treated for the indicated time points (lo. ex. represents prolonged exposure of the blots; sh. ex. represents short exposure). (**C**) CEM cells were pre-incubated with zVad and Necrostatin-1 (Nec-1) for 30 min and then treated with DMSO (Veh), DMF (50 μM), PMX464 (PMX, 1 μM), etoposide (Eto, 50 μM) or NBDII (50 μM) for 16 h. Immunoprecipitation (IP) was performed with antibodies against caspase-8 (α-C8). As negative control, DMF treated cells were incubated with goat IgG (left). Complex formation of caspase-8 (C8) and RIPK1 was analyzed by Proximity ligation assay (PLA). Depicted are z-stacks; Hoechst (grey), C8/RIPK1 (red), and Brightfield (**B**); scale bar, 10 μm (right). (**D**) SeAx cells were treated with DMF (50 μM) for the indicated time points. Interaction of RIPK1 and caspase-8 was determined by PLA (red), Hoechst (grey). Shown are representative confocal microscopy images and mean fluorescence intensities (MFI) (n = 3). (**E**) Knock-down of *Birc3* (cIAP2) results in ripoptosome-dependent cell death. siRNA-transfected CEM cells were analyzed by qPCR (left), specific cell death (middle), and PLA (right) (n = 3). Complexes of caspase-8 (C8) and RIPK1 are depicted in red and indicated with a white arrow, Hoechst (grey), Brightfield (B); scale bar, 10 μm. (D, E) Error bars represent standard deviation. Statistics were calculated using Student’s t-test (*p < 0.05; **p < 0.005; ***p < 0.001).

NFκB inhibition by DMF caused cell death in HH, SeAx and CEM cells in a dose- and time-dependent manner (Supplementary Fig. S3A). Since Trx1 inhibition by DMF triggered ROS accumulation, we tested whether ROS is the source for cell death. However, cell death mediated by DMF was independent of ROS. Trolox diminished ROS accumulation upon DMF treatment (Fig. 1C), but failed to rescue DMF-induced cell death (Supplementary Fig. S3B). Thus, ROS and Iron-dependent cell death can be excluded^29^. Instead, cell death mediated by DMF was facilitated by caspase-8 and -3 activation (Fig. 3A; Supplementary Figure S3C). Importantly, activation of caspase-8 and -3 was independent of extrinsic death ligands. DMF failed to stimulate mRNA expression of CD95L, TNFα and TRAIL in malignant T-cells (Supplementary Fig. S4A). In addition, treatment with inhibitors for CD95L (APG101), TNFα (Enbrel) and TRAIL (TRAIL-Fc), either alone or in combination, had no effect on cell death induced by DMF (Supplementary Fig. S4B,C).

Since no extrinsic ligands were involved in caspase activation upon NFκB inhibition, we set out to determine whether an intrinsic cell death platform called the ripoptosome facilitates PCD. Ripoptosome formation is blocked by IAPs. Downregulation of IAPs upon DMF administration (Fig. 2D) coincided with elevated caspase-8 activity (Fig. 3A). It was reported that RIPK1 is negatively regulated by caspase-8 and FADD once incorporated in the ripoptosome complex. Active caspase-8 cleaves RIPK1 after D324, thus, promoting apoptosis but inhibiting necroptosis^30^. Consistent with DMF-induced kinetics of caspase-8 activation (Fig. 3A) RIPK1 was cleaved upon DMF administration (Fig. 3B; Supplementary Fig. S4D). Abrogation of caspase activity by zVad application rescued RIPK1 cleavage in DMF treated cells suggesting complex formation of caspase-8 with RIPK1. As expected, loss of cIAP2 expression was not influenced upon zVad administration (Fig. 3B).

To elucidate ripoptosome assembly in response to DMF, we tested interaction of caspase-8 with RIPK1 and FADD using the proximity ligation assay. Fluorescent signals from interaction of caspase-8 with RIPK1 or FADD were detected in response to DMF (Supplementary Fig. S4E). Accordingly, DMF treatment triggered co-immunoprecipitation of caspase-8 with FADD and RIPK1 in CEM and SeAx cells (Fig. 3C, Supplementary Fig. S4F). Complex formation of caspase-8 and RIPK1 occurred 4–8 h after DMF exposure in SeAx cells and was most robust after 24 h in CEM cells (Fig. 3D; Supplementary Fig. S4G). To further establish that loss of NFκB activity triggered ripoptosome formation additional inhibitors of the NFκB signaling pathway (PMX464 and NBDII) were tested. Similar to DMF, PMX464 blocks NFκB activity by suppressing Trx1 (Supplementary Fig. S2A)^28^. NBDII (NEMO binding domain peptide) antagonizes the interaction of NEMO with the IKK complex and, therefore, suppresses NFκB activation. Administration of PMX464 and NBDII revealed that inhibition of the NFκB signaling pathway resulted in ripoptosome formation. Etoposide, which causes depletion of IAPs and subsequent ripoptosome-dependent cell death^14^, was used as positive control (Fig. 3C, Supplementary Fig. S4F).

Since DMF-mediated inhibition of NFκB resulted in decreased cIAP2 expression most efficiently, we tested whether knock-down of *Birc3* led to ripoptosome-dependent cell death. siRNA-mediated knock-down of about 60% resulted in moderate ripoptosome assembly and subsequent PCD (Fig. 3E). Thus, our data indicate that antagonizing NFκB activity induces ripoptosome formation, which is mediated by cIAP2 downregulation.

### Activation of the mitochondrial pathway is required for ripoptosome assembly and ripoptosome-dependent cell death

IAPs are negative regulators of ripoptosome formation. cIAP2 was downregulated in response to DMF-dependent NFκB suppression, which resulted in ripoptosome assembly. To evaluate whether additional IAPs were involved in DMF-mediated assembly of the ripoptosome, we tested the role of cIAP1 and XIAP. cIAP1 and XIAP mRNA expression was not altered upon DMF administration (Supplementary Fig. S5A). However, protein expression was impaired in response to DMF after 4–8 h suggesting an additional mechanism that led to decreased protein levels of cIAP1 and XIAP (Supplementary Fig. S5B).

**Figure 4:**
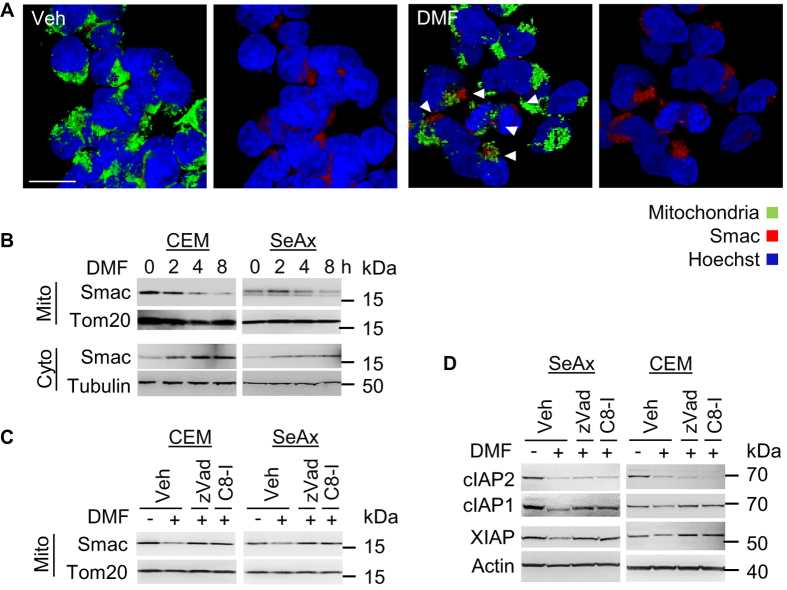
DMF application induces Smac release. (**A**) Shown are representative z-stacks of cells treated with DMSO (Veh) or DMF (50 μM) for 8 h. Paraformaldehyde fixed cells were stained for Smac (red), mitochondria (green), Hoechst (blue), and analyzed by confocal microscopy; arrows indicate Smac release; scale bar, 10 μm. (**B**) Mitochondria were isolated from DMF (50 μM) treated cells as indicated. Cytoplasmic and mitochondrial fractions were analyzed by Western blot. (**C**) DMSO (Veh), zVad, and caspase-8 inhibitor (C8-I) incubated cells were left untreated or stimulated with DMF (50 μM) for 8 h. Shown are Western blot analyses of isolated mitochondria (Mito). (**D**) Protein lysates of cells treated as described in (**C**) were analyzed for expression of cIAP2, cIAP1, XIAP and Actin.

Caspase-8 can activate downstream effector caspases such as caspase-3 directly. Simultaneously, caspase-8 can induce Bid cleavage. Bid cleavage was observed 4–8 h after DMF administration (Supplementary Fig. S5C). Cleaved Bid results in MOMP and subsequent release of pro-apoptotic molecules such as cytochrome c and Smac^31^. Since Smac has been reported to promote degradation of IAPs, we tested whether Smac was released by mitochondria in response to NFκB inhibition. Indeed, DMF-mediated Smac release occurred most robustly after 4–8 h, which coincided with a decrease in cIAP1 and XIAP protein levels (Fig. 4A,B and Supplementary Fig. S5B). Release of Smac was caspase-dependent since inhibition of caspase-8 resulted in decreased Smac translocation from mitochondria (Fig. 4C). Moreover, inhibition of caspase-8 led to recovery of decreased protein expression levels of cIAP1 and XIAP but not cIAP2 (Fig. 4D). This is consistent with previous results showing that cIAP2 expression was regulated by NFκB activity (compare Fig. 2). Thus, downregulation of cIAP2 in response to DMF results in Smac release from the mitochondria and in degradation of XIAP and cIAP1.

**Figure 5:**
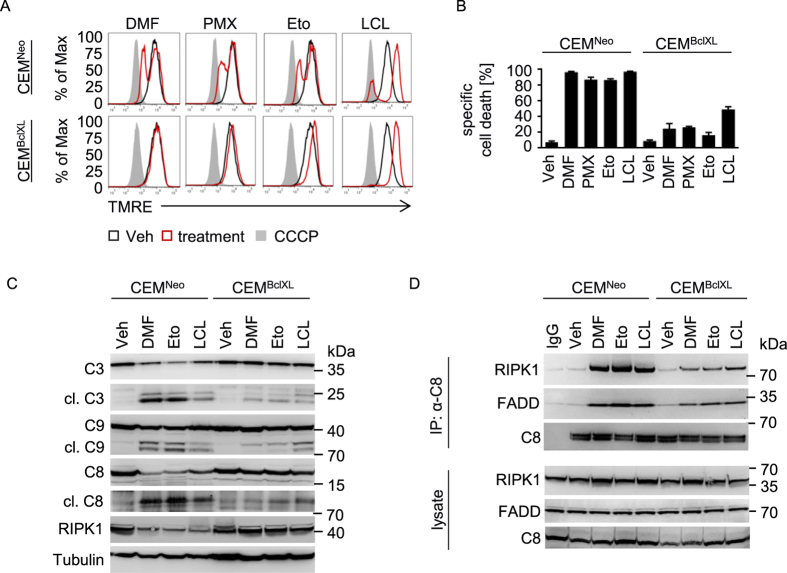
A mitochondrial amplification loop promotes ripoptosome formation. (**A**) CEM^Neo^ or CEM^BclXL^ cells were treated with DMF (50 μM), PMX464 (PMX, 1 μM), LCL161 (LCL, 20 μM), or etoposide (Eto, 50 μM) for 8 h (red). DMSO was used as vehicle control (black). CCCP was used as positive control (grey). MOMP was assessed by staining with TMRE. (**B**) Specific cell death was analyzed in cells stimulated with DMF (50 μM), PMX464 (PMX, 1 μM), LCL161 (LCL, 20 μM), or etoposide (Eto, 50 μM) for 18 h (n = 3). (**C**) CEM^Neo^ or CEM^BclXL^ cells were treated with DMSO (Veh), DMF (50 μM), etoposide (Eto, 50 μM), or LCL161 (LCL, 20 μM) for 18 h. Lysates were subjected to Western blot and analyzed for caspase-3 (C3), −9 (C9), −8 (C8), RIPK1 and Tubulin. (**D**) Mitochondrial amplification loop promotes ripoptosome formation. Cells were pre-incubated with zVad and Nec-1, then stimulated as in (**C**) for 24 h. IP was performed with antibodies against caspase-8 (α-C8). (**B**) Error bars represent standard deviation.

Next, we evaluated whether Smac release and subsequent degradation of IAPs was necessary for ripoptosome-dependent cell death. Therefore, we used Bcl_XL_ overexpressing cells (CEM^BclXL^) and compared them with control CEM cells (CEM^Neo^). Bcl_XL_ inhibits MOMP-dependent release of mitochondrial contents such as Smac. In CEM^Neo^ cells DMF diminished mitochondrial membrane potential. In contrast, overexpression of Bcl_XL_ abolished MOMP (Fig. 5A). Interestingly, Bcl_XL_ overexpression diminished ripoptosome-dependent cell death suggesting that involvement of mitochondria is critical for DMF-mediated PCD (Fig. 5B). In concert, Bcl_XL_ suppressed cleavage of caspase-9 and caspase-3 in response to DMF. Surprisingly, DMF administration resulted in reduced caspase-8 cleavage in CEM^BclXL^ cells. In addition, caspase-8-dependent RIPK1 cleavage was rescued by Bcl_XL_ suggesting that mitochondria are critical for ripoptosome formation and subsequent cell death (Fig. 5C). Indeed, Bcl_XL_ diminished complex formation of RIPK1/FADD/caspase-8 in response to DMF indicating an essential mitochondrial amplification loop, which promotes ripoptosome assembly (Fig. 5D).

To further characterize PCD we compared the effect of DMF with PMX464, etoposide, and LCL161. LCL161 is a Smac mimetic, which induces assembly of the ripoptosome by depleting IAPs^32^. Similar to DMF treatment, induction of MOMP in response to PMX464, etoposide, or LCL161 was diminished by Bcl_XL_ (Fig. 5A). PCD was rescued by Bcl_XL_ upon PMX464 or etoposide administration. However, LCL161 treated CEM^BclXL^ cells revealed moderate inhibition of PCD (Fig. 5B). This is in line with caspase cleavage and complex formation. Assembly of the ripoptosome and caspase cleavage upon LCL161 administration was only moderately inhibited suggesting that the Smac mimetic is less dependent on MOMP-mediated Smac release (Fig. 5C,D). In contrast, Bcl_XL_ diminished complex formation and caspase cleavage in response to etoposide (Fig. 5C,D) indicating that both, DMF and etoposide require mitochondria for ripoptosome-dependent cell death. Thus, overexpression of Bcl_XL_ inhibits mitochondrial Smac release and subsequent degradation of IAPs (*e.g.* cIAP1 and XIAP) in response to DMF. Collectively, these data suggest that reduction of cIAP2 in response to DMF-induced NFκB inhibition results in moderate ripoptosome formation. However, to enhance cell death, sufficient assembly and activation of the ripoptosome requires a caspase-8-dependent mitochondrial amplification loop characterized by MOMP and Smac-induced degradation of cIAP1 and XIAP.

### Ripoptosome-dependent cell death in human malignant T-cells occurs in an apoptotic and necroptotic manner

CTCL, including its leukemic variant Sézary syndrome, is characterized by a CD4^+^, CD45RO^+^ and CD8^−^ phenotype and an elevated expression of constitutively active NFκB accompanied by resistance to PCD^2^,^20^,^33^. DMF is a potent inhibitor of NFκB activity and was reported to induce apoptosis in stimulated human T-cells^34^.

**Figure 6:**
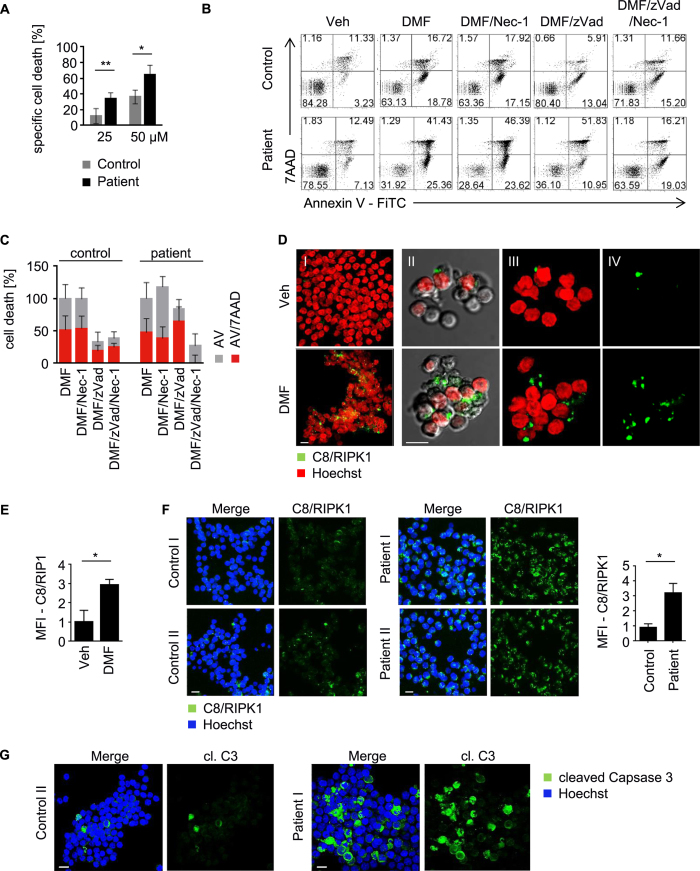
DMF-dependent assembly of the ripoptosome augments cell death in human Sézary cells. (**A**) Specific cell death of T-cells from healthy donors (control) and Sézary patients (n = 5) treated with DMF (50 μM) or vehicle control was assessed. (**B**) Isolated T-cells from CTCL-positive or -negative donors were stimulated as indicated, stained with AnnexinV-FiTC and 7AAD, and analyzed by flow cytometry. (**C**) Quantification of AnnexinV-FiTC (AV) and AnnexinV-FiTC/7AAD (AV/7AAD) positive cells normalized to DMF treatment (n = 5). (**D**) T-cells from Sézary patients (n = 2) were stimulated with vehicle or DMF (50 μM) for 24 h. Complex formation of caspase-8 (C8) and RIPK1 (green) was determined by PLA, Hoechst (red); shown are representative immunofluorescences; scale bar: 10 μm (I), z-stack, merge of C8/RIPK1 and Hoechst; (II) single plane image, merge of C8/RIPK1, Hoechst and Brightfield; (III) z-stack, merge of C8/RIPK1 and Hoechst; (IV) z-stack, C8/RIPK1. (**E**) Quantification of mean fluorescence intensities acquired from representative images as depicted in (**D**), (n = 3). (**F**) Ripoptosome formation in DMF treated Sézary patients. Patient I and II were treated with 480 mg/d DMF for 26 and 10 weeks, respectively. Complex formation of RIPK1 and caspase-8 (C8) was determined by PLA in isolated CD4^+^ T-cells from DMF treated patients and healthy donors (Control I and II) and is depicted in green; nuclear staining with Hoechst is depicted in blue; shown are representative immunofluorescence analyses from z-stacks; scale bar, 10 μm; (left). Mean fluorescence intensities of PLA signals were quantified (n = 4); (right). (**G**) Depicted are z-stacks from healthy donor (Control II) and DMF treated patient (patient I). Isolated CD4^+^ T-cells were stained for cleaved caspase-3 (cl. C3, green) and Hoechst (blue); scale bar, 10 μm. (**A,E,F**) Error bars represent standard deviation. Statistics were calculated using Student’s t-test (*p < 0.05; **p < 0.005; ***p < 0.001).

CD4^+^ T-cells were isolated from Sézary patients (Supplementary Table 1), which revealed elevated levels of phosphorylated NFκB, cIAP2 and cFLIP compared to healthy controls (Supplementary Fig. S6A). To evaluate the impact of DMF treatment on malignant human T-cells, we first measured specific cell death of DMF-treated primary resting and malignant CD4^+^ T-cells. NFκB inhibition by DMF led to increased PCD in T-cells isolated from Sézary patients (Fig. 6A). To determine the mode of cell death upon NFκB inhibition, apoptosis was distinguished from necroptosis by Annexin V-FiTC/7AAD uptake (Fig. 6B,C). Interestingly, zVad protected resting CD4^+^ cells from PCD but only marginally inhibited DMF-induced cell death in malignant T-cells. Here, zVad administration promoted necroptosis in almost all patient samples since zVad reduced the Annexin V-FiTC positive population, but did not affect the appearance of double-positive cells. Only the combination of zVad with the RIPK1 inhibitor Necrostatin-1 (Nec-1) protected cells from DMF-induced PCD (Fig. 6B,C; Supplementary Fig. S6B). Similarly, inhibition of NFκB signaling by DMF, PMX464 or NBDII resulted in necroptotic cell death in CEM cells (Supplementary Fig. S6C,D). Thus, inhibitors of NFκB signaling can mediate necroptosis *via* the ripoptosome. LCL161^35^, which induces necroptosis in a variety of cell lines when treated together with zVad, was included as control (Supplementary Fig. S6C).

Consistent with the notion that malignant T-cells can die in an apoptotic or necroptotic manner, a similar mode of PCD was observed in stimulated T-cells (Supplementary Fig. S6E,F). PCD in response to DMF-dependent NFκB inhibition was elevated in stimulated T-cells (Supplementary Fig. S6E). In contrast to unstimulated T-cells, which exclusively die by apoptosis, PCD in stimulated T-cells was characterized by apoptosis and/or necroptosis upon caspase inhibition (Supplementary Fig. S6F), suggesting that stimulated as well as malignant T-cells share a similar phenotype revealing NFκB dependency. Furthermore, PCD was induced by ripoptosome formation because caspase-8 was complexed with RIPK1 upon DMF application in Sézary cells (Fig. 6D,E). Importantly, Sézary patients orally treated with DMF revealed substantial ripoptosome formation compared to untreated healthy donors (Fig. 6F). Moreover, caspase-3 cleavage was augmented in a majority of isolated CD4+ cells from patient II indicating that DMF induces PCD in Sézary patients *via* the ripoptosome (Fig. 6G). Thus, application of DMF in Sézary patients may represent a promising approach for treatment of CTCL and other NFκB-dependent tumors.

## Discussion

NFκB is a promising target for treatment of lymphoproliferative diseases *e.g.* CTCL. So far a variety of NFκB Inhibitors have been tested such as Velcade^®^, curcumin and nonsteroidal anti-inflammatory therapeutics^36^,^37^,^38^. However, these drugs reveal limited therapeutic responses or are too toxic for clinical application. In contrast, DMF is a FDA approved drug used for treatment of psoriasis and multiple sclerosis displaying minor side effects. Previously, it has been shown that DMF application suppresses tumor growth and metastasis in a mouse CTCL xenograft model^20^. Therefore, it is of particular interest to investigate the inhibitory activity of DMF for the development of a novel class of improved NFκB inhibitors.

Here we show that therapeutic intervention targeting the NFκB signaling pathway in malignant T-cells results in cell death that is mediated by the ripoptosome. DMF has been reported to inhibit NFκB nuclear translocation, which leads to diminished production of pro-inflammatory cytokines and adhesion molecules^39^,^40^. Mechanistically, it was shown that DMF interferes with intracellular thiols^40^. Trx1 is a cellular redox scavenger, which is active when its reactive cysteine residues (C32, C35) are reduced^41^. Thioredoxin reductase 1 (TrxR1) can reduce oxidized C32 and C35 and, therefore, controls the activity of Trx1. The interaction of Trx1 and thioredoxin reductase is regulated by C73 another cysteine in vicinity to the postulated interaction side of both proteins^24^,^26^. Cancer cells often reveal elevated expression of active Trx1 and TrxR1, which maintains a reduced nuclear redox state providing an excellent environment for transcriptional activity^42^. We show that DMF modifies Trx1 at C73 leading to an inhibition of Trx1. NFκB is a redox-regulated transcription factor. Trx1 reduces NFκB in the nucleus and, thereby, enhances DNA-binding activity. Although it is possible that DMF influences other proteins apart from NFκB, depletion of IAPs coincided with the gene-induction profile upon NFκB inhibition. Loss of IAPs is associated with PCD triggered by DMF. Our experiments revealed that DMF-dependent induction of PCD was independent of death receptor engagement. Further, NFκB inhibition did not result in altered expression of death ligands such as TNF, CD95L and TRAIL in lymphoma cells. This indicates that the complex consisting of caspase-8, RIPK1 and FADD formed in response to NFκB inhibition is the intrinsic cell death platform called ripoptosome. In addition, our data indicate that a mitochondrial amplification loop improves ripoptosome formation in the T-ALL cell line CEM. This amplification loop is characterized by ripoptosome-dependent caspase-8 activity responsible for Smac-dependent degradation of IAPs and, thus, increased ripoptosome assembly (Supplementary Fig. S7). Therefore, it is likely that the extent of ripoptosome formation depends on induction of the mitochondrial amplification loop. In turn the amount of ripoptosome formation determines the extent of apoptotic and necroptotic cell death: Treatment with LCL161 (less dependent on the mitochondrial amplification loop) results in strong necroptotic cell death when caspase-8 is inhibited. In contrast, DMF or etoposide induce a weak ripoptosome initially. Caspase-8 activity is required to enhance assembly of the ripoptosome *via* mitochondria. Accordingly, once caspase-8 activity is blocked by zVad, the mitochondrial amplification loop is lacking and ripoptosome formation is not augmented. Thus, induction of necroptosis upon zVad administration is less profound compared to LCL161 treated cells (Supplementary Fig. S8).

In summary, our observations show that inhibition of Trx1 results in diminished NFκB transcriptional engagement. DMF-dependent suppression of NFκB leads to downregulation of cIAP2 and cFLIP, which negatively regulate ripoptosome formation. Ripoptosome assembly is associated with caspase-8 activity, which promotes the mitochondrial amplification loop. This molecular mechanism provides important new information for prospective therapeutic interventions in cancer. Depending on the cellular context, elevated expression of anti-apoptotic Bcl-2 family members protecting mitochondria might dampen the ripoptosome-dependent response upon DMF administration. Thus, a combination of ripoptosome-inducing agents, such as Trx1 antagonists and inhibitors of Bcl-2 family members, might improve the anti-tumor responses.

Of note, non-stimulated T-cells exclusively die by apoptosis. In contrast, pre-activated as well as malignant CD4^+^ T-cells, which share a similar phenotype, can die by necroptosis once caspase-8 activity is inhibited. This is in agreement with a report that necroptosis occurs as a result of antigen receptor-mediated activation in caspase-8 deficient T-cells^13^. The additional cell death pathway in form of necroptosis in pre-activated or malignant T-cells could point to an important checkpoint for T-cell homeostasis. Moreover, necroptosis has been implied to release massive amounts of damage-associated molecular patterns (DAMPs)^43^. Hence, necroptotic cell death of CTCL might trigger a strong inflammatory anti-tumor response.

Finally, our results demonstrate that inhibition of NFκB *via* Trx1 leads to ripoptosome formation and subsequent cell death in malignant T-cells. DMF exhibits only minor side effects compared to other anti-cancer drugs including Smac mimetics and therapeutic agents directly inhibiting NFκB. Therefore, targeting the Trx1 system including Trx1 and TrxR1 is a promising therapeutic approach. Accordingly, DMF represents a novel substance class of small-molecule inhibitors to treat lymphoproliferative diseases and other tumors with constitutive NFκB activation.

## Materials and Methods

### Chemicals

Chemicals were obtained from Sigma unless otherwise indicated. Cells were treated with either zVad (25 μM or 50 μM, Bachem), Necrostatin-1 (Nec-1, 50 μM), caspase-8 inhibitor (50 μM, Merck), or respective combinations 30 min prior stimulation with either DMF, Etoposide (Biovision), LCL161 (Active Biochem), NBDII (Merck), PMX464 (Tocris) or TNFα (10 ng/ml) and Cycloheximide (Chx, 10 μg/ml). Cells were incubated with 5 μM carbonyl cyanide m-chlorophenyl hydrazone (CCCP) 10 min before measurement.

### Cells

SeAx, HH and CEM cells were cultured in RPMI medium supplemented with 10% FCS and 0.1 mg/ml streptomycin or gentamycin. CEM^BclXL^ cells over-expressing Bcl_XL_ and and CEM^Neo^ control cells were selected with Neomycin^44^. The CTCL cell line HH and the ALL cell line CEM were originally from American Type Culture Collection (ATCC). SeAx cells were kindly provided by S. Eichmüller (German Cancer Research Center, Germany). Cells were tested by the cell contamination control unit of the DKFZ^45^.

### Antibodies

Anti-caspase-8 monoclonal antibody C15 recognizes the p18 subunit of caspase-8. Anti-FLIP monoclonal antibody NF6 recognizes the N-terminal part of c-FLIP. Anti-FADD monoclonal antibody 1C4 recognizes the C-terminal part of FADD^46^. Antibodies against Caspase-3, caspase-9, p65, Phospho-NFκB p65 (Ser536), cIAP1, cIAP2, XIAP, Bcl_XL_, and Smac were obtained from Cell Signaling. Antibodies specific for Bcl-2 were purchased from Santa Cruz Biotechnology, RIPK1 from BD Bioscience, Trx and Tom20 from Abcam, β-Actin from Genetech and Tubulin from Sigma.

### Cell death assays and Luciferase activity assays

Cell death was analyzed by FSC/SSC or Annexin V-FiTC/7-Aminoactinomycin D (7AAD) staining using flow cytometry as described previously^47^. Specific cell death was calculated^29^. To determine mitochondrial membrane potential (∆Ψ_m_), cells were incubated with 50 μM Tetramethylrhodamine ethyl ester (TMRE, Life Technologies) for 30 min. Caspase activity was measured using Caspase-Glo assay system (Promega) according to manufacturer’s recommendations.

### Thioredoxin activity assay

The Thioredoxin Activity Fluorescent Assay Kit (Cayman) uses fluorescence to measure active Trx in microtiter plates and was performed according to manufacturer’s recommendations. The method is based on the reduction of insulin by reduced Trx.

### Immunoprecipitation and pull-down assays

For co-immunoprecipitation of caspase-8 bound proteins, 1–2 × 10^7^ cells were pre-stimulated with zVad (25 μM) 30 min prior to treatment with indicated substances. Caspase-8 was precipitated according to manufacturer’s instructions (Promega). Equal amounts of eluates were analyzed by Western blot.

Two complementary biotinylated oligonucleotides containing NFκB binding sites were annealed and 1 μg of double stranded oligonucleotides was incubated with 400 μg of nuclear extracts in 500 μl binding buffer (12% glycerol, 12 mM HEPES, pH 7.9, 4 mM Tris, pH 7.9, 150 mM KCl, 1 mM EDTA, 1 mM DTT, 0.1 μg/μl poly (dI-dC) and 0.5 μg/μl BSA). Protein complexes bound to each oligonucleotide were precipitated with preblocked streptavidin beads (Life Technologies), and analyzed by Western blot. For oligonucleotide sequences see Supplementary Information.

For pull-down of free thiols, cells were lysed with low pH buffer (50 mM 2-(N-morpholino) ethanesulfonic acid, pH 6.5), containing 1% Triton X-100 (v/v),100 mM NaCl, and Complete-Mini protease inhibitor cocktail (Roche). Proteins containing free thiols incubated with biotinylated iodoacetamide (100 μM, BIAM, Life technologies) were precipitated with 50 μl Streptavidin beads pre-adsorbed with 1 mg/ml BSA. Proteins with accessible thiol groups were analyzed by Western blot.

### Immunofluorescence

Immunofluorescent stainings and acquisition of mean fluorescence intensities (MFIs) were performed as described^48^. Primary antibodies (Smac, FADD (Abcam), caspase-8 (Santa Cruz), RIPK1 (BD Bioscience)), fluorophore-conjugated secondary antibodies and Hoechst33342 (Life Technologies) were diluted 1:50–1:100. For staining of mitochondria MitoTracker Deep Red (Life technologies) was added to secondary antibodies.

To visualize protein interactions a Proximity Ligation Assay (PLA) was performed according to manufacturer’s recommendations (Sigma). Immunofluorescent stainings were analyzed by confocal microscopy (LSM710, Zeiss).

### Lymphocyte separation

Human peripheral blood leukocytes were purified as described^29^. Then, T-cells were sorted with the CD4^+^ T-Cell Isolation Kit II according to manufacturer’s instruction (Miltenyi Biotec). The study was conducted according to ethical guidelines of the DKFZ and the Helsinki Declaration, and was approved by the ethics committee II of the Ruprecht-Karls-University of Heidelberg.

## Additional Information

**How to cite this article:** Schroeder, A. *et al*. Targeting Thioredoxin-1 by dimethyl fumarate induces ripoptosome-mediated cell death. *Sci. Rep.* **7**, 43168; doi: 10.1038/srep43168 (2017).

**Publisher's note:** Springer Nature remains neutral with regard to jurisdictional claims in published maps and institutional affiliations.

## Supplementary Material

Supplementary Information
