# Supplementary material for: Targeting Thioredoxin-1 by dimethyl fumarate induces ripoptosome-mediated cell death
Source: Sci Rep. 2017 Feb 24;7:43168. doi: 10.1038/srep43168 (PMC5324128; doi:10.1038/srep43168)
Supplement: Supplementary Information [file srep43168-s1.pdf]

SUPPLEMENTARY INFORMATION

Targeting Thioredoxin-1 by dimethyl fumarate induces ripoptosome-mediated cell death

Anne Schroeder, Uwe Warnken, Daniel Röth, Karel D. Klika, Diana Vobis, Andrea Barnert, Fatmire Bujupi, Tina Oberacker, Martina Schnölzer, Jan P. Nicolay, Peter H. Krammer, and Karsten Gülow,

SUPPLEMENTARY INFORMATION

Table of contents:

- Supplementary Figure Legends
- Supplementary Data
- Supplementary Materials and Methods
- Supplementary References
- Supplementary Figures (1-8)
- Supplementary Table 1

Supplementary Figure Legends

Supplementary Figure S1. DMF inhibits Trx1 activity. (A) Reaction of GSH and DMF to provide the glutathione-dimethyl fuamrate adduct. The atom numbering and the newly created asymmetric center denoted by an asterisk in the product are indicated. Thus, for the product, two disastereomers are realized. (B) CEM cells were treated with vehicle control or DMF (25µM) for 2 h. Proteins were separated under non-reducing conditions and samples were subjected to MS analysis. Identified peptides in the Trx1 protein sequence are highlighted in red. The protein sequence coverage was 51 %. (C) In the DMF-treated sample, a fraction of the Trx1 peptide CMPTFQFFK was identified as monomethyl succinylated with an individual Mascot peptide score of 39. (D) MSMS spectrum of the monomethylsuccinylated Trx peptide CMPTFQFFK (left panel) and the corresponding MSMS peptide fragments annotated by Mascot software (right panel). The peptide was identified with an individual Mascot peptide score of 39.

Supplementary Figure S2. DMF inhibits NFκB signaling pathway. (A) CEM cells were treated with DMF (50 µM) or PMX464 (PMX, 1µM) for 2h. Reduced proteins were pulled down using biotinylated iodoacetamide (BIAM) and probed for phosphorylated NFκB (P-p65). (B) Altered DNA-binding of NFκB in response to DMF. HH and SeAx cells were treated with DMF (50 µM) for 4 h. Oligo pull down was performed for indicated target genes (top). Band intensities were quantified from 3 independent experiments and normalized to negative control (bottom). (C) Quantitative real-time PCR (qPCR) of *Bcl-2* was performed on RNA derived from cells treated with DMF (50 µM) for the indicated periods of time. (D) Oxidative stress and phosphorylation results in activation and nuclear translocation of NFκB. Nuclear NFκB requires reduction to permit DNA binding. DMF-dependent monomethyl succinylation of Trx1 (Trx) prevents reduction of cysteines in NFκB. Thus, transcriptional activity of NFκB on target genes such as *Birc3* and *Cflar* is suppressed. (B, C) Error bars represent standard deviation.

Supplementary Figure S3. DMF-induced cell death requires caspase activity. (A) DMF induces apoptotic cell death. HH, SeAx and CEM cells were treated with DMF (black square) in a dose-dependent manner (0, 30, 50, 70, 100 μM) for 24 h (left); or time-dependent manner (0, 2, 4, 8, 12, 24 h) with 50 μM (right). Specific cell death was determined by flow cytometry. DMSO (white square) was used as vehicle control. (B) Cell death triggered by DMF is ROS independent. HH, SeAx and CEM cells were treated with DMSO (Veh), MMF, DMF or Trolox with concentrations indicated for 16 h. Specific cell death was determined by flow cytometry. (C) Caspase-3 (C3) cleavage and activity of HH and SeAx cells treated with DMF (50 µM) was determined by Western blot, activity assay (left) and immunofluorescence (IF, right) for the indicated time points. For IF, Hoechst is depicted in grey, caspase-3 activity in red and caspase-8 activity in green; scale bar, 100 μM. (A, B, C) Error bars represent standard deviation.

Supplementary Figure S4. DMF-mediated cell death depends on ripoptosome formation and not on extrinsic ligands (TNFα, CD95L, TRAIL). (A) QPCR analysis of indicated genes. RNA was isolated from cells treated with DMF (50 µM) for indicated time points. (B) DMF-induced cell death is independent of extrinsic pathway. Specific cell death was determined from cells stimulated with DMF (50 µM, black) or vehicle (white) and incubated with indicated concentrations of APG101, Enbrel, or TRAIL-Fc overnight. (C) Combination of inhibitors of extrinsic ligands failed to inhibit DMF-dependent apoptosis. Cells were treated with DMSO (Veh, grey), DMF (black), or a combination of DMF, APG101 (500 µg/ml), Enbrel (30 µM) and TRAIL-Fc (10 µM) (blue). (D) Shown is a Western blot analysis of HH and SeAx cells treated with DMF (50 µM) in a time-dependent manner and probed against RIPK1 and actin. (E) Proximity ligation assay (PLA) of CEM cells treated with DMF (50 µM) for 24 h. Complex formation of caspase-8 and RIPK1 (red) or caspase-8 and FADD (green). Hoechst (grey); scale bar, 10 µm. (F) SeAx cells were treated with DMF (50 µM), etoposide (Eto, 50 µM), NBDII (50 µM) or vehicle control (Veh,

DMSO) for 18 h. Interaction of RIPK1 and FADD with caspase-8 (C8) were determined by co-Immunoprecipitation by pulling down C8 (α-C8). As negative control, DMF treated cells were incubated with goat IgG. (G) CEM cells were stimulated with DMF (50 µM) in a time-dependent manner as indicated. Caspase-8 IP was performed as described in figure 3C. (A, B, C) Error bars represent standard deviation.

Supplementary Figure S5. Requirement of mitochondria for ripoptotosome-dependent cell death. (A) Quantitative real-time PCR of indicated genes was performed using RNA derived from cells treated with DMF (50 µM) for 2, 4, and 8 h. (B) HH, SeAx and CEM cells were incubated with DMF (50 µM) for the indicated time points. Cell lysates were analyzed by Western blot. (C) DMF induces Bid cleavage in a time-dependent manner. Shown is a Western blot of DMF (50 µM) treated cells probed for Bid and Actin. (A) Error bars represent standard deviation.

Supplementary Figure S6. DMF induces apoptosis and necroptosis. (A) $CD4^+$ T-cells isolated from healthy controls (n = 3) and Sézary patients (n = 2) were subjected to Western blot and probed with antibodies as indicated. (B) $CD4^+$ T-cells from Sézary patients (n = 3) were treated with zVad or Necrostatin-1 (Nec-1) as indicated 30 min before administration of DMF (50 µM) or DMSO (Veh). The next day cells were stained with AnnexinV-FiTC as well as 7AAD and analyzed by flow cytometry. (C) CEM cells were treated with zVad or Necrostatin-1 (Nec-1) as indicated. DMSO (Veh), DMF, PMX464 (PMX), NBDII or LCL161 were added with concentrations indicated for 12 h. Then, cells were stained with AnnexinV-FiTC/7AAD and analyzed by flow cytometry. (D) Quantification of AnnexinV-FiTC (AV) and AnnexinV-FiTC/7AAD (AV/7AAD) positive cells treated with DMF (50 µM), zVAd and Nec-1 as described in (B), and normalized to DMF treatment (n = 10). (E) Primary T-cells from healthy donors, stimulated with PHA and cultured with IL-2 for 6d or left unstimulated, were treated with DMF as indicated. Specific cell death was determined by flow

cytometry. (F) Resting and active primary T-cells treated as indicated, stained with AnnexinV-FiTC and 7AAD, and analyzed by flow cytometry. (D, E) Error bars represent standard deviation.

Supplementary Figure S7. Ripoptosome assembly in response to loss of NFκB activity. DMF, PMX464 and NBDII suppress the NFκB signaling pathway. Inactivation of NFκB leads to downregulation of anti-apoptotic genes including cIAP2 and cFLIPs (cFLIP$_L$ and cFLIP$_S$). Loss of cIAP2 results in ripoptosome formation. Assembly of the ripoptosome leads to apoptotic and necroptotic cell death. Necroptosis occurs when caspase-8 activity is inhibited by zVad. Apoptosis is induced upon inhibition of NFκB and is caspase dependent. Full ripoptosome activation requires the mitochondrial cell death pathway. Caspase-8 cleaves Bid resulting in MOMP-dependent Smac release. Release of Smac from mitochondria into the cytosol promotes degradation of IAPs including cIAP1 and XIAP, which further amplifies ripoptosome formation and activation in a feed-forward loop. LCL161 promotes degradation of IAPs directly and is therefore less dependent on mitochondria.

Supplementary Figure S8. A mitochondrial amplification loop promotes ripoptosome assembly in response to inhibitors of NFκB signaling. DMF administration results in reduced cIAP2 expression and subsequent assembly of the ripoptosome. Activation of caspase-8 promotes cleavage of RIPK1, a major regulator of necroptosis. In addition, active caspase-8 induces the mitochondrial amplification loop, which further enhances ripoptosome formation and subsequent apoptosis. When caspase-8 is inactive RIPK1 is not inhibited and the mitochondrial amplification loop is missing. Therefore, degradation is less profound resulting in minor PCD in form of necroptosis (left). Smac mimetics such as LCL161 induce degradation of IAPs including cIAP1, cIAP2 and XIAP and are therefore less dependent on the mitochondrial amplification loop. Thus, administration of Smac mimetics leads to strong necroptotic cell death when caspase-8 is inhibited (right).

Supplementary Table 1. Pathological characteristics of Sèzary patients (n = 5) compared to healthy status (Norm). Therapy involves extracorporal photohoresis (ECP) and interferon-α (IFN-α) application.

Supplementary Data

NMR Data

The reaction of DMF with glutathione was clear from the appearance of new sp^3-hybridized CH and CH_2 groups. The identity of the glutathione–dimethyl fumarate adduct was established by partial assignment of the ^1H and ^{13}C NMR chemical shifts (δ) while the integrity of the attachment of the glutathione and DMF units was proven unequivocally by HMBC correlations across the thioether linkage. As expected, the creation of a new asymmetric center in the adduct realizes two disastereomers and thus two sets of NMR signals for the product are observed. For the ^1H NMR signals, a number of them were overlapped with signals of unreacted glutathione which was still present in the reaction mixture. Thus, they could only be reported as overlapped multiplets and in these cases, δ values were taken by necessity from the 2D spectra.

Glutathione–dimethyl fumarate adduct. ^1H NMR δ: 4.628 (dd, J_{H12B} = 8.82 , J_{H12A} = 5.02 Hz, H-5), 4.614 (dd, J_{H12B} = 8.09 , J_{H12A} = 5.29 Hz, H-5), 3.95 and 3.92 (ho ol m, 2 × H-2), 3.81 (ho ol m, 2 × H-14), 3.776 and 3.764 (2 × 3H, s, 2 × CH_3O-16 or CH_3O-17), 3.743 (t, J_{H9A} = J_{H9B} = 6.38 Hz, 2 × H-10), 3.698 (6H, s, 2 × CH_3O-16 or CH_3O-17), 3.230 (dd, J_{H12B} = −14.10, J_{H5} = 4.90 Hz, H-12A), 3.180 (dd, J_{H12B} = −13.96, J_{H5} = 5.36 Hz, H-12A), 3.016 (dd, J_{H12A} = −13.98, J_{H5} = 8.50 Hz, H-12B), 2.91 (ol, H-12B), 2.94 (ho ol m, 2 × H-15A), 2.79 (ho m, 2 × H-15B), 2.54 (ol m, 2 × H-8), 2.15 (ol m, 2 × H-9). ^{13}C NMR δ: 177.39 and 177.27 (2 × C-7), 176.59 and 176.54 (2 × C-16 or C-17), 176.01 and 176.00 (2 × C-11), 175.82 and 175.80 (2 × C-1), 175.52 and 175.49 (2 × C-16 or C-17), 174.79 and 174.77 (2 × C-4), 57.00 (2 × C-10), 56.24 and 55.70 (2 × C-5), 55.81 and 55.77 (2 × CH_3O-16 or CH_3O-17), 55.18 and 55.16 (2 × CH_3O-16 or CH_3O-17), 45.29 and 44.47 (2 × C-14), 44.34 and 44.33 (2 × C-2), 38.8 and 38.5 (2 × C-15), 36.29 and 35.93 (2 × C-12), 34.53 and 34.50 (2 × C-8), 29.37 and 29.32 (2 × C-9).

Supplementary Materials and Methods

NanoLC-MS/MS, data processing and protein database search

1 x 10^6 CEM cells treated with or without DMF (4 h, 25 µM) were lysed with RIPA buffer, protein lysates were separated in a 1D-PAGE and in-gel digested as described before.[1] For Thioredoxin identification, four gel slices excised in a mass range from 10-20 kDa were analyzed applying 1 h gradients for nanoLC-MS/MS as described previously.[2]

MS raw data were processed using Xcalibur software (Thermo Fisher Scientific) and protein sequence database searches were performed using Mascot search engine 2.4 (Matrix Science) applying SwissProt database (SwissProt 2015_08, 549008 sequences, 195692017 residues). Peptide mass tolerance for database searches was set to 5 ppm and fragment mass tolerance to 0.4 Da. Cysteine carbamidomethylation was set as variable modification as well as cysteine 2-dimethyl succinylation, 2-monomethyl succinylation, succinylation and oxidation of methionine. One missed cleavage site in case of incomplete trypsin hydrolysis was allowed. Proteins were considered as identified if at least two unique peptides had an individual ion score exceeding Mascot identity threshold (ion score cut-off: 30). Protein identification refers to false discovery rate (FDR) < 1% and match probability of $p < 0.01$.

siRNA transfection and knock down

CEM cells were transfected with non-targeting control #1 (Ambion, #4390843) or *Birc3* siRNA (Ambion, #4392420) using the Amaxa protocol for Jurkat cells. 1x10^6 cells were incubated with 1 µM siRNA and co-transfected with GFP control [0.5 µg/µl]. The next day, cells were analyzed by qPCR and flow cytometry. For cell death GFP positive cells were gated and then analyzed by FSC and SSC. For proximity ligation assay (PLA) CEM cells were incubated with Accell siRNA for *Birc3* (Dharmacon, #E-004099-00) or non-solencing control (Dharmacon, #D-001910-01-20) according to manufacturer's recommendations. Upon knock down cells were subjected to PLA and analyzed by confocal microscopy.

Mitochondria isolation

For subcellular fractionation of functional mitochondria 2 x 10^7 cells were lysed. Mitochondria were isolated by magnetic field separation using the Mitochondria isolation kit (Miltenyi Biotech).

qRT-PCR analysis

Total RNA was isolated using the RNeasy kit (Qiagen). Reverse transcription and quantitative reverse transcription-PCR (RT-PCR) was performed as described.[3] Primers were obtained from Qiagen.

Oligo-pull-down primer sequences

Oligonucleotides used for pull-down assays with NFκB sites include promoter regions of *Cflar* (AGGGGAATTCC), *Birc2* (TGGAATTCCCC), *Birc3* (TGGAAATCCCC), *Xiap* (CCGGGACCCTCCC), *Bcl2L1* (AAGGGACTTCTCA), and *Bcl2* (AGGGAACTCCCCC). The oligonucleotide GATCCATTAGGGGATGCCCCTCAT was used as positive control.

Caspase activity

For Immunofluorescence staining of caspase activity, Image-iT LIVE Green caspase-3 Detection Kit (Life Technologies) was used according to manufacturer's instructions. Caspase activity was analyzed at the confocal microscope (LSM700 or LSM710, Zeiss).

NMR acquisition

NMR spectra were acquired using a Bruker Avance III NMR spectrometer equipped with a 5-mm, normal-configuration probe with z-gradient capability at a field strength of 9.4 T operating at 400.1 and 100.6 MH*z* for ^1H and ^{13}C nuclei, respectively, at 303 K in $D_2O–CD_3OD$. Pulse widths were calibrated following the described protocol.[4] The chemical shifts of ^1H and ^{13}C nuclei are reported relative to TSP (δ = 0 ppm for both ^1H and ^{13}C) incorporated as an internal standard. The

chemical shifts of ^{1}H nuclei are reported to three decimal places when signals were interpretable by first-order analysis or to two decimal places when signals were taken indirectly from 2D spectra due to overlap or were reported only as higher-order multiplets. The chemical shifts of ^{13}C nuclei are reported to two decimal places when signals were observable directly or one decimal place when signals were taken indirectly from 2D spectra. General NMR experimental and acquisition details for 1D ^{1}H, ^{13}C and DEPT and standard gradient-selected 2D COSY, HSQC and HMBC spectra and routine chemical shift assignment using 2D NMR have been previously described.[5-8]

Preparation of GSH–DMF adduct

Glutathione in the reduced form (ca. 5 mg) was taken up in ca. 0.3 ml of D_2O and to this was added DMF (ca. 7.5 mg) in ca. 0.3 ml of CD_3OD. The heterogenous mixture was then heated in a water bath at 41.5 °C for several hours to provide a homogeneous solution with the glutathione–dimethyl fumarate adduct as a minor component in the presence of unreacted glutathione and DMF. NMR analysis was conducted on this reaction mixture in situ.

Proximity ligation assay and immunofluorescent stainings of tumor sections

To determine protein interactions in cells by Proximity Ligation Assay (PLA), cells were fixed with 2% PFA, permeabilized in PBST^{++} (PBS^{++}, 0.1% Triton X100), and blocked in 1% BSA in PBS^{++} (1x PBS, 1 mM $MgCl_2$, 0.1 mM $CaCl_2$). Cells were incubated with primary antibodies (FADD (Abcam), caspase-8 (Santa Cruz), RIPK1 (BD Bioscience)), fluorophore-conjugated secondary antibodies and Hoechst33342 (Life Technologies) diluted 1:50 – 1:100 in 0.2% BSA in PBS^{++}. Secondary antibodies conjugated to oligonucleotides (PLA probe MINUS and PLA probe PLUS) were diluted 1:5 in 1x diluent (Sigma), supplemented with Hoechst33342 (Life Technologies) and incubated with ligation stock to hybridize oligonucleotides to PLA probes when in close proximity according to manufacturer’s instructions. Upon adding amplification solution containing

polymerase, nucleotides and fluorescently labeled oligonucleotides, cells were mounted with Mowiol. Stainings of tissue sections were performed as described previously.[9]

Equipment and settings

Western Blots were visualized at the Vilber Lourmat using the ChemiCapt software. Images were cropped and processed with the Microsoft Office Picture Manager software.

Microscopic images were obtained at the LSM700 or LSM710 (Carl Zeiss, Jena, Germany). ZEN microscope software was used to acquire images. Images were recorded in 12-bit mode using same settings. Data processing was performed with ZEN microscope software 2.1 and Adobe Photoshop CS3 using same settings among experiments.

Figure 3C:

x:512, y:512, z:9; 12-bit; scan mode: stack; objective: Plan-Apochromat 63x/1.40 Oil DIC M27; lasers: track1: 405 nm – 2%, track2: 488 nm – 14%; filters: track1: 410-585, track2: 493-630; beam splitters: track1: MBS:Plate, MBS_InVis:MBS-405, FCS EF:BlackPlate, FCS DBS:NFT490; track2: MBS:MBS488, MBS_InVis:Plate, DBS1:Mirror, FCS EF:Black plate, FCS DBS:NFT490

Figure 3D:

x:512, y:512, z:23; 12-bit; scan mode: stack; objective: EC Plan-Neofluar 40x/1.30 Oil DIC M27; lasers: track1: 488 nm – 40%, track2: 405 nm – 2.4%; filters: track1: 493-1000, track2: SP 490; beam splitters: track1: MBS405/488/555/639, DBS1:492 nm; track2: MBS:MBS405/488/555/639, DBS1:502 nm

Figure 3E:

x:512, y:512, z:11; 12-bit; scan mode: stack; objective: Plan-Apochromat 63x/1.40 Oil DIC M27; lasers: track1: 405 nm – 2%, track2: 488 nm – 15%; filters: track1: 410-585, track2: 493-630; beam splitters: track1: MBS:Plate, MBS_InVis:MBS-405, DBS1:Mirror, FCS EF:BlackPlate, FCS DBS:NFT490;

track2: MBS:MBS488, MBS_InVis:Plate, DBS1:Mirror, FCS EF:Black plate, FCS DBS:NFT490

Figure 4A:

x:512, y:512, z:13; 12-bit; scan mode: stack; objective: Plan-Apochromat 63x/1.40 Oil DIC M27; lasers: track1: 633 nm – 7.5%, track2: 488 nm – 15%, track3: 405 nm – 2.9%; filters: track1: 638-755, track2: 494-630, track3: 410-507; beam splitters: track1: MBS:MBS488/561/633, MBS_InVis:Plate, DBS:Mirror, FCS EF:BlackPlate; track2: MBS:MBS488, MBS_InVis:Plate, DBS1:Mirror, FCS EF:Black plate; track3: MBS:Plate, MBS_InVis:MBS-405, DBS1:Mirror, FCS EF:Black plate

Figure 6D:

x:512, y:512, z:20; 12-bit; scan mode: stack; objective: EC Plan-Neofluar 40x/1.30 Oil DIC M27; lasers: track1: 488 nm – 13%, track2: 405 nm – 13%; filters: track1: 493-1000, track2: SP 490; beam splitters: track1: MBS405/488/555/639, DBS1:492 nm; track2: MBS:MBS405/488/555/639, DBS1:496 nm

Figure 6F:

x:512, y:512, z:16; 12-bit; scan mode: stack; objective: Plan-Apochromat 63x/1.40 Oil DIC M27; lasers: track1: 488 nm – 13%, track2: 405 nm – 2%; filters: track1: 0-630, track2: 0-508; beam splitters: track1: MBS405/488/555/639, DBS1:630 nm; track2: MBS:MBS405/488/555/639, DBS1:508 nm

Figure 6G:

x:512, y:512, z:16; 12-bit; scan mode: stack; objective: Plan-Apochromat 63x/1.40 Oil DIC M27; lasers: track1: 488 nm – 1.8%, track2: 405 nm – 2.4%; filters: track1: 0-630, track2: 0-508; beam splitters: track1: MBS405/488/555/639, DBS1:630 nm; track2: MBS:MBS405/488/555/639, DBS1:508 nm

Supplementary References

1 Schleich, K. *et al.* Stoichiometry of the CD95 death-inducing signaling complex: experimental and modeling evidence for a death effector domain chain model. *Molecular cell* **47**, 306-319, doi:10.1016/j.molcel.2012.05.006 (2012).

2 Warnken, U., Schleich, K., Schnolzer, M. & Lavrik, I. Quantification of High-Molecular Weight Protein Platforms by AQUA Mass Spectrometry as Exemplified for the CD95 Death-Inducing Signaling Complex (DISC). *Cells* **2**, 476-495, doi:10.3390/cells2030476 (2013).

3 Kiessling, M. K. *et al.* Inhibition of constitutively activated nuclear factor-kappaB induces reactive oxygen species- and iron-dependent cell death in cutaneous T-cell lymphoma. *Cancer research* **69**, 2365-2374, doi:10.1158/0008-5472.CAN-08-3221 (2009).

4 Klika, K. D. The Application of Simple and Easy to Implement Decoupling Pulse Scheme Combinations to Effect Decoupling of Large Values with Reduced Artifacts. *Int J Spectr* **2014**, 1-9, doi:10.1155/2014/289638 (2014).

5 Virta, P. *et al.* Synthesis, characterisation and theoretical calculations of 2,6-diaminopurine etheno derivatives. *Org Biomol Chem* **3**, 2924-2929, doi:10.1039/b505508c (2005).

6 Klika, K. D. *et al.* Unexpected formation of a spiro acridine and fused ring system from the reaction between an N-acridinylmethyl-substituted thiourea and bromoacetonitrile under basic conditions. *J Org Chem* **66**, 4416-4418 (2001).

7 Balentova, E. *et al.* Stereochemistry, tautomerism, and reactions of acridinyl thiosemicarbazides in the synthesis of 1,3-thiazolidines. *J Heterocycl Chem* **43**, 240–249, doi:10.1002/jhet.5570430318 (2006).

8 Mäki, J., Tähtinen, P., Kronberg, L. & Klika, K. D. Restricted rotation/tautomeric equilibrium and determination of the site and extent of protonation in bi-imidazole nucleosides by multinuclear NMR and GIAO-DFT calculations. *J. Phys. Org. Chem.* **18**, 240-249, doi:10.1002/poc.840 (2005).

9 Nicolay, J. P. *et al.* Dimethyl fumarate restores apoptosis sensitivity and inhibits tumor growth and metastasis in CTCL by targeting NFkappaB. *Blood*, doi:10.1182/blood-2016-01-694117 (2016).

A

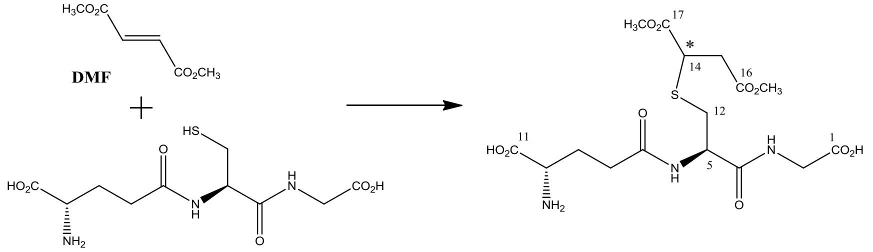

Glutathione

Glutathione-DMF adduct

B

1	MVKQIESK**TA**	**FQEALDAAGD**
21	**K**LVVVDFSAT	**WCGPCKMIKP**
41	**FFHSLSEK**YS	NVIFLEVDVD
61	DCQDVASECE	VK**CMPTFQFF**
81	**K**KGQK**VGEFS**	**GANKEKLEAT**
101	**INELV**	

C

Mr(expt)	Mr(calc)	Score	Peptide
1335.6322	1335.6306	96	K.TAFQEALDAAGDK.L
1257.6830	1257.6816	62	K.EKLEATINELV.-
907.4413	907.4399	54	K.VGEFSGANK.E
1204.5417	1204.5409	45	K.CMPTFQFFK.K + Carbamidomethyl (C)
1293.5427	1293.5410	39	K.CMPTFQFFK.K + 2-monomethylsuccinyl (C); Oxidation (M)
1204.5426	1204.5409	37	K.CMPTFQFFK.K + Carbamidomethyl (C)
1478.7579	1478.7592	35	K.MIKPFFHSLSEK.Y + Oxidation (M)
1220.5364	1220.5359	37	K.CMPTFQFFK.K + Carbamidomethyl (C); Oxidation (M)
1462.7641	1462.7642	33	K.MIKPFFHSLSEK.Y

D

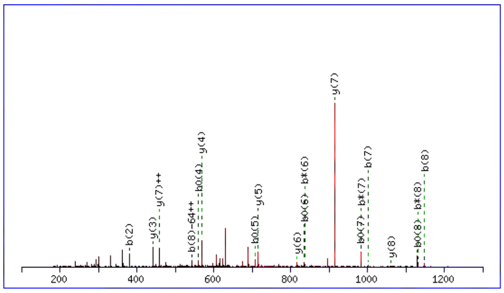

#	b	b*	b^0	Seq.	y	#
1	234.0431			C		9
2	*381.0785*			M	*1061.5125*	8
3	478.1312			P	*914.4771*	7
4	579.1789		561.1683	T	*817.4243*	6
5	726.2473		708.2368	F	*716.3766*	5
6	854.3059	837.2794	836.2953	Q	*569.3082*	4
7	*1001.3743*	984.3478	983.3638	F	*441.2496*	3
8	*1148.4427*	1131.4162	1130.4322	F	294.1812	2
9				K	147.1128	1

Supplementary Figure 1

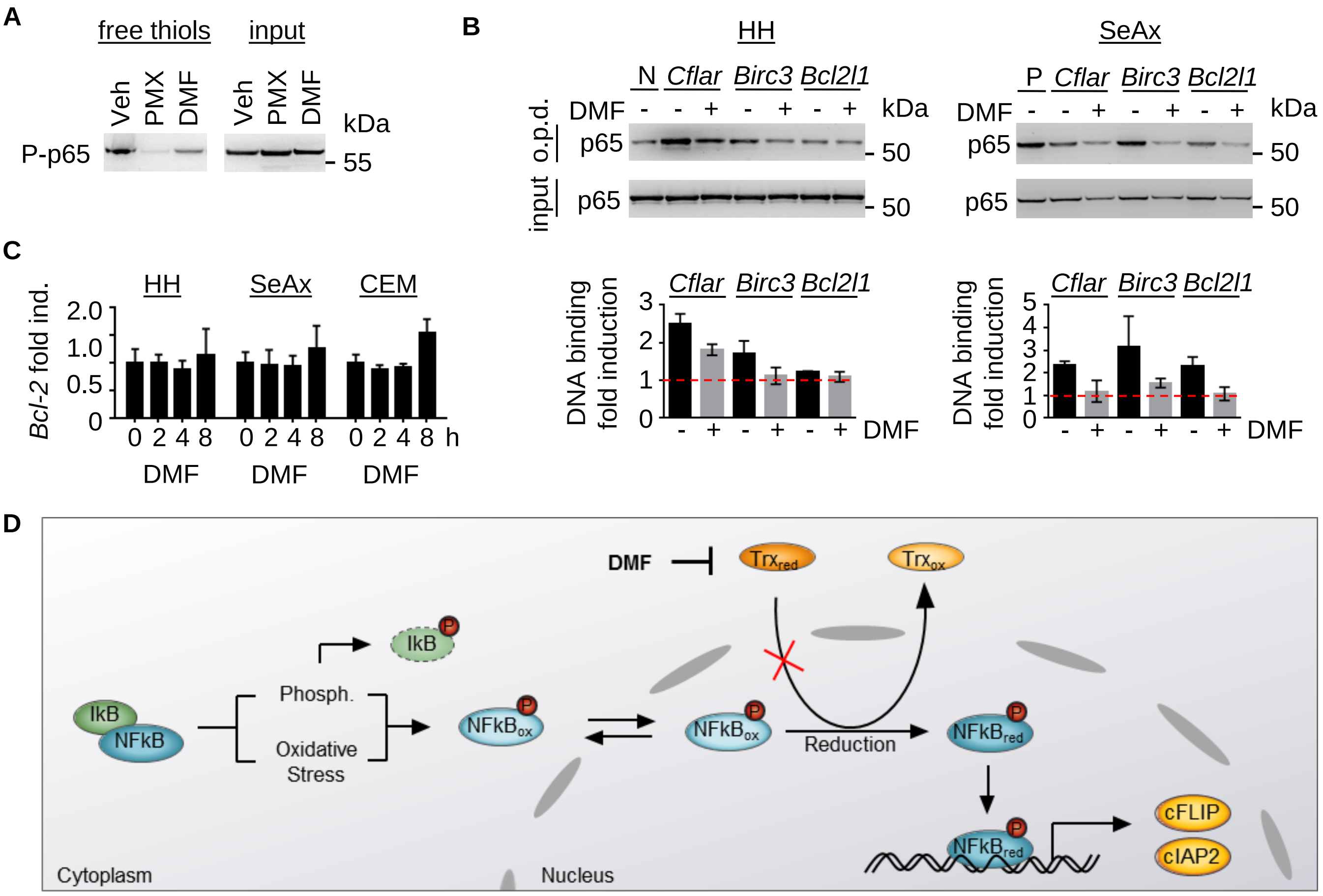
A
free thiols
input
Veh
PMX
DMF
Veh
PMX
DMF
kDa
P-p65
55
B
HH
SeAx
N
Cflar
Birc3
Bcl2l1
P
Cflar
Birc3
Bcl2l1
DMF
kDa
o.p.d.
p65
50
input
p65
50
C
HH
SeAx
CEM
Bcl-2 fold ind.
2.0
1.0
0.5
0
0 2 4 8
0 2 4 8
0 2 4 8 h
DMF
DMF
DMF
DNA binding
fold induction
DMF
D
DMF
Trxred
Trxox
IkB
Phosph.
Oxidative
Stress
NFkB
NFkBox
NFkBred
Reduction
cFLIP
cIAP2
Cytoplasm
Nucleus

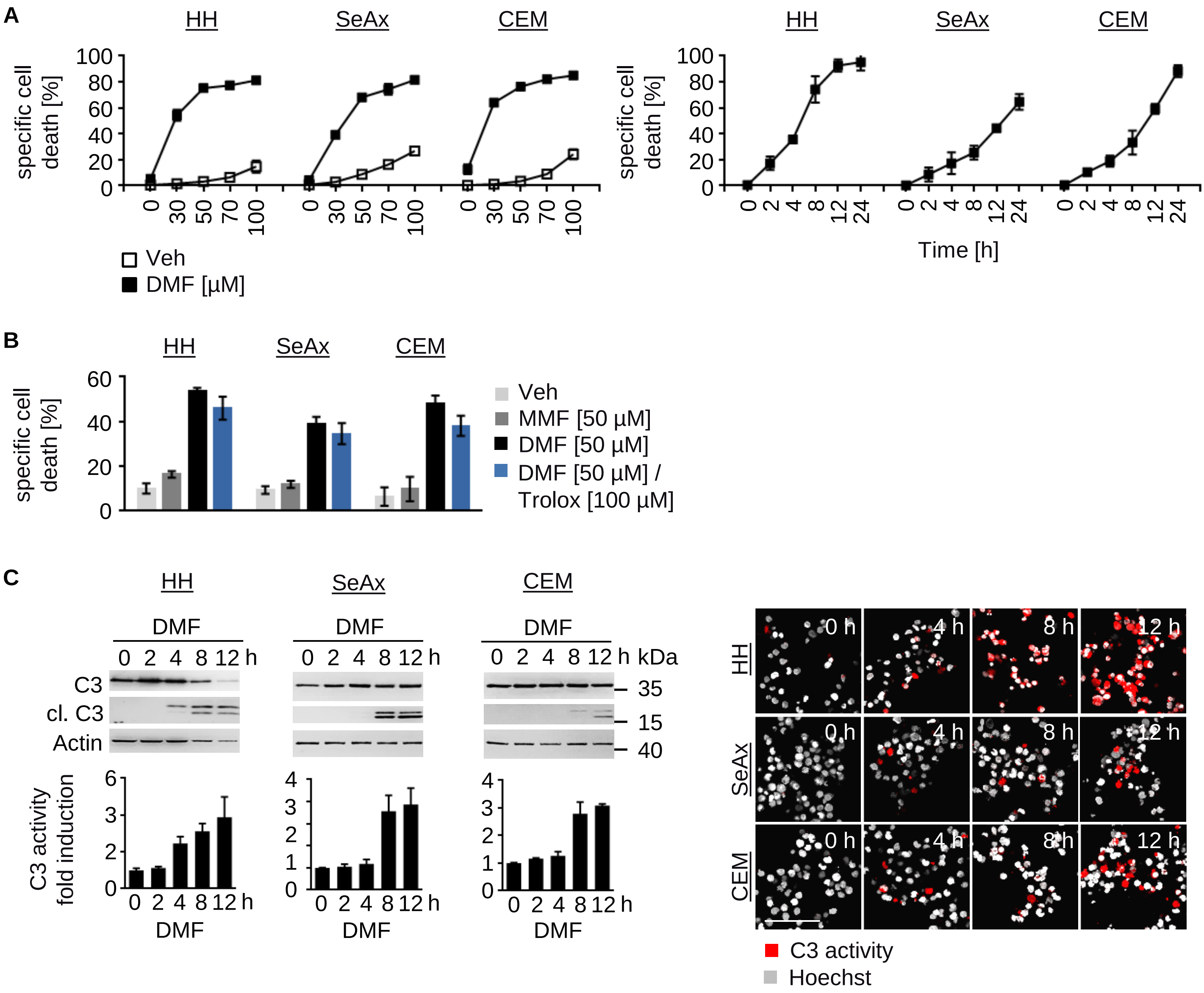
A
HH
SeAx
CEM
specific cell death [%]
100
80
60
40
20
0
0
30
50
70
100
Veh
DMF [µM]
0
2
4
8
12
24
Time [h]
B
specific cell death [%]
60
40
20
Veh
MMF [50 µM]
DMF [50 µM]
DMF [50 µM] / Trolox [100 µM]
C
DMF
0 2 4 8 12 h
kDa
C3
cl. C3
Actin
35
15
40
C3 activity fold induction
6
3
2
1
0
4
0 h
4 h
8 h
12 h
C3 activity
Hoechst

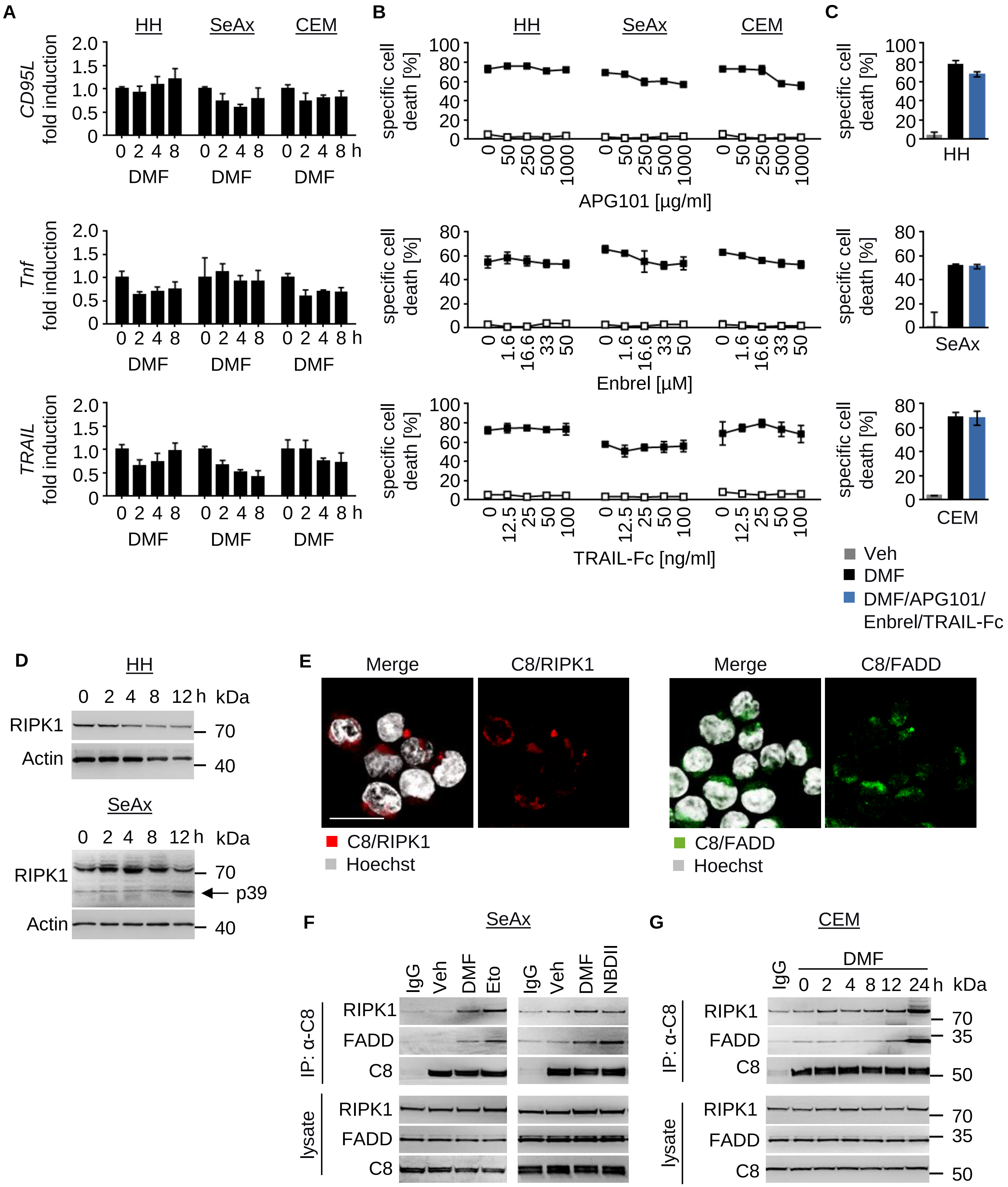

Supplementary Figure 4

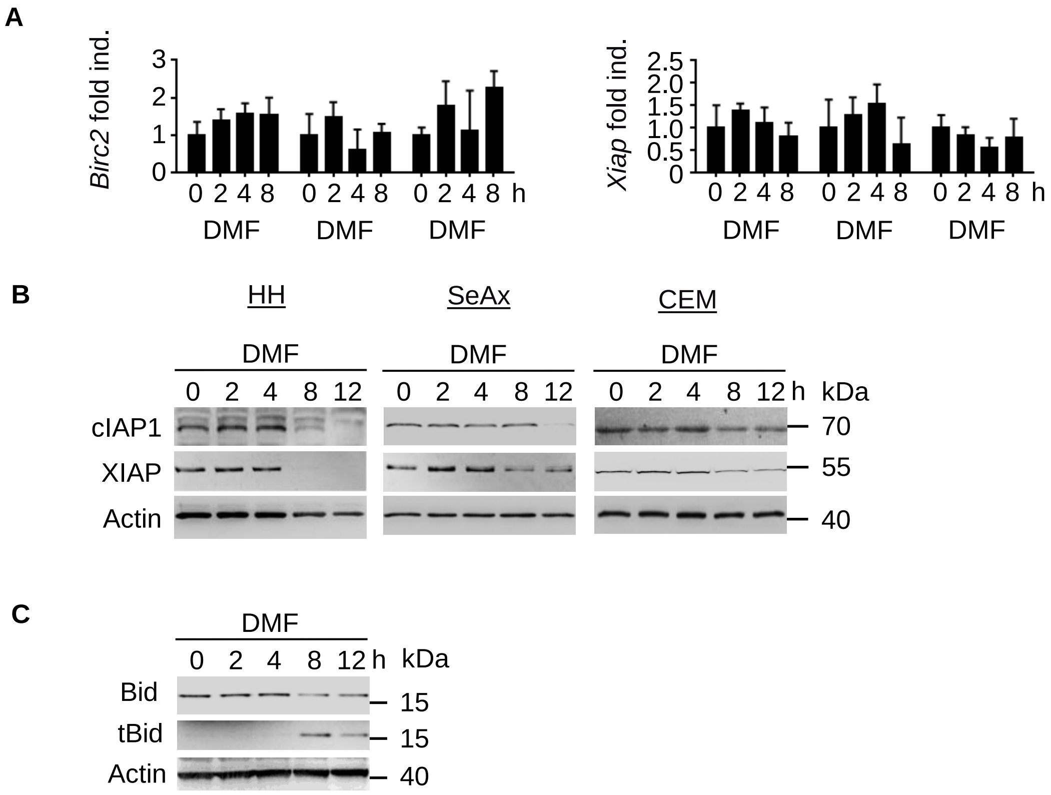

Supplementary Figure 5

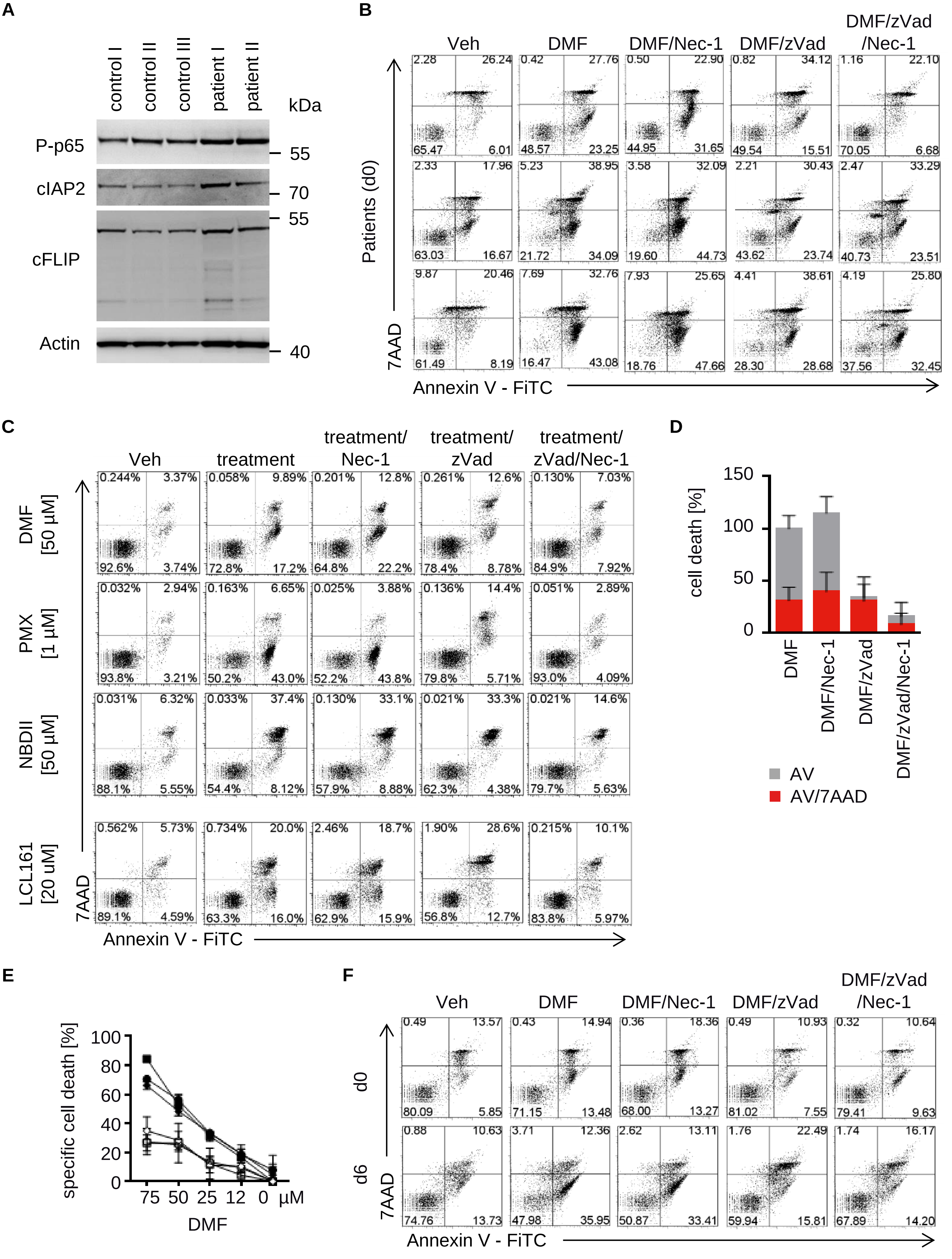

Supplementary Figure 6

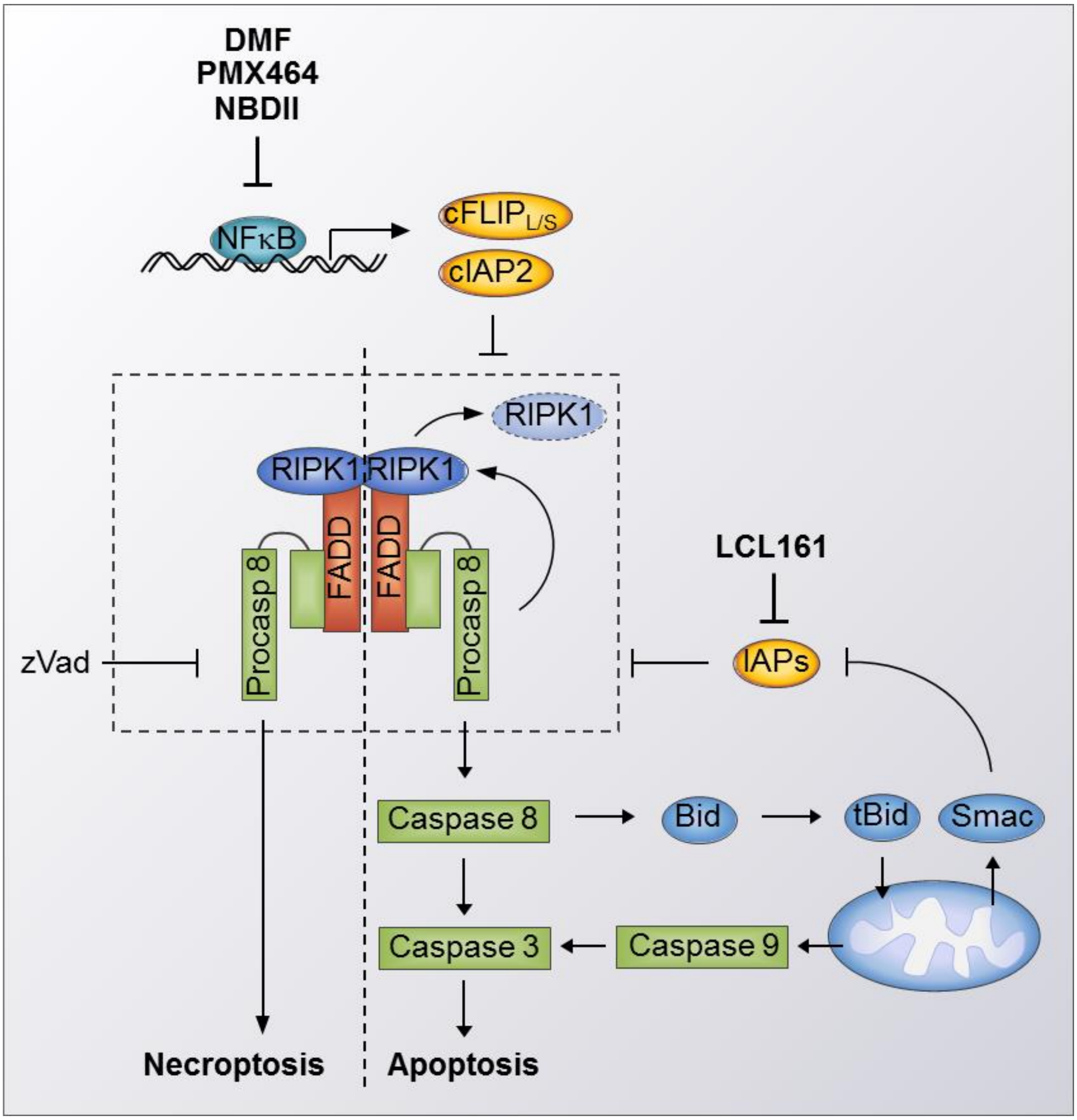
DMF
PMX464
NBDII
NFκB
cFLIP L/S
cIAP2
RIPK1
RIPK1
RIPK1
FADD
FADD
Procasp 8
Procasp 8
zVad
LCL161
IAPs
Caspase 8
Bid
tBid
Smac
Caspase 3
Caspase 9
Necroptosis
Apoptosis

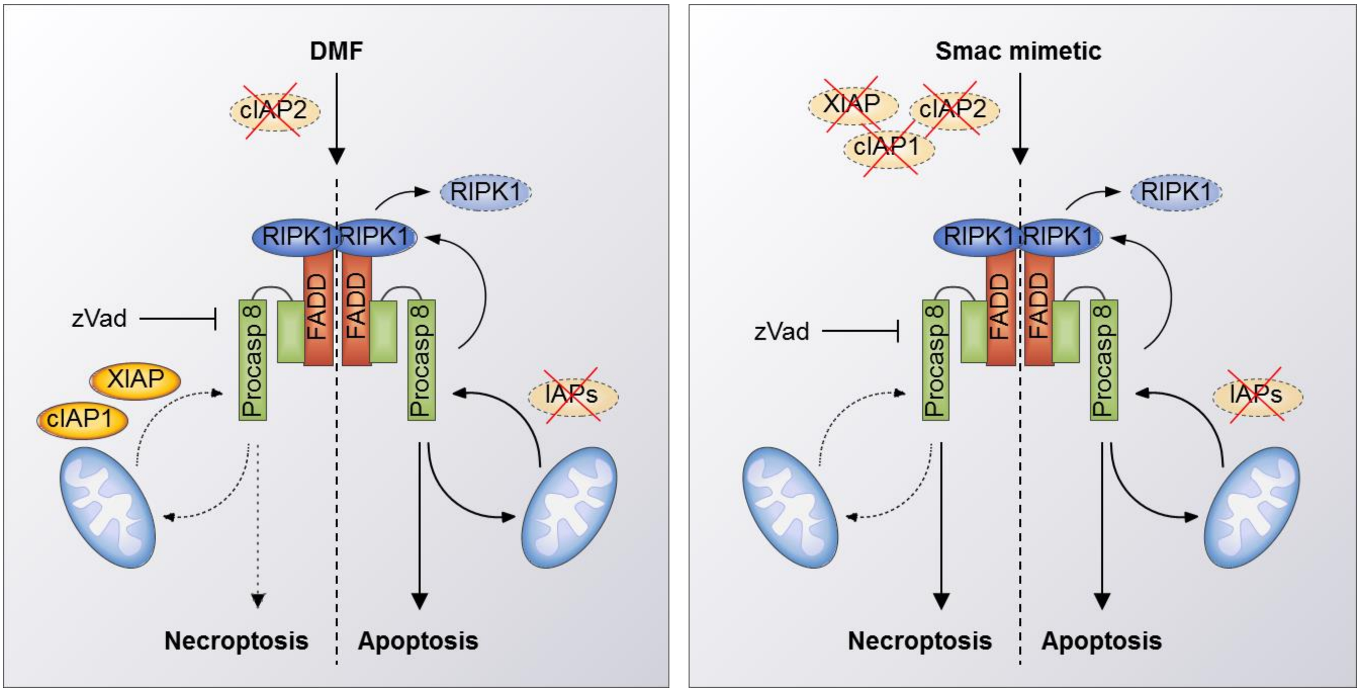

Supplementary Figure 8

Supplementary Table 1

Patient	gender, age	TNM	Leukocytes/nl	T lymphocytes	CD4 T lymphocytes/µl	Sezary cells/µl	CD4/CD8 cell ratio	skin involvement	% skin involved	duration of therapy
1	m, 73	T4NxM0B2	7.03	35.6%	1672	1359	13.8	mild	30	4 months
2	m, 68	T4NxM0B2	13.49	42.8%	5483	3172	20.7	severe	90	1 month
3	m, 68	T4NxM0B2	7.98	33.6%	2235	1810	17.7	severe	100	4 months
4	m, 68	T4NxM0B2	15.89	46.0%	5125	2768	30.2	severe	90	2 months
5	m, 68	T4NxM0B2	6.47	29.1%	1037	684	18.1	severe	80	5 months
Physiological Values			4,2 - 10,2	60 - 83%	528 - 1495	< 1000	1 - 2,8			

T = Tumor
N = Lymphnode
M = Metastasis
B = Blood
